# 1p-Enh-regulated CYP4B1 alleviates NNK-induced heart failure and lung cancer via the STAT3 pathway

**DOI:** 10.1371/journal.pone.0331471

**Published:** 2025-09-09

**Authors:** Li Dai, Jun Hu, Ye Yang, Bingjun Qian, Xianglin Zuo

**Affiliations:** 1 Department of Basic Medical Sciences, Jiangsu Medical College, Yancheng, Jiangsu, PR China; 2 Biobank of Jiangsu Cancer Hospital, Jiangsu Institute of Cancer Research & The Affiliated Cancer Hospital of Nanjing Medical University, Nanjing, Jiangsu, PR China; Affiliated Hospital of Nantong University, CHINA

## Abstract

Heart failure (HF) and lung cancer (LC) often coexist, yet their shared molecular mechanisms are unclear. We analyzed transcriptome data from the NCBI Gene Expression Omnibus (GEO) database (GSE141910, GSE57338) to identify 346 HF‑related differentially expressed genes (DEGs), then combined weighted gene co-expression network analysis (WGCNA) pinpointed 70 hub candidates. Further screening of these 70 hub candidates in TCGA lung cancer cohorts via LASSO, Random Forest, and multivariate Cox regression suggested *CYP4B1* as the only independent prognostic marker. Subsequent ROC analysis validated *CYP4B1*’s diagnostic power in both HF and LC (AUC > 0.80). Immune-cell infiltration analysis demonstrated that high *CYP4B1* expression correlated with increased infiltration of M2 macrophages. Experiments revealed *CYP4B1* downregulation in angiotensin II (Ang II)-induced cardiomyocytes (AC-16) and LC cells (A549 & H1703). *CYP4B1* overexpression attenuated angiotensin-II–induced cardiac hypertrophy and inhibited the migration, invasion, and proliferation of LC cells. Mechanistic studies revealed that *CYP4B1* suppresses the JAK-STAT3 signaling, and we identified a novel distal enhancer, 1p‑Enh, that regulates *CYP4B1* expression via chromatin looping. Additionally, prolonged exposure to the tobacco carcinogen NNK suppressed 1p‑Enh activity and downregulated *CYP4B1* expression. These findings demonstrate the critical role of the NNK‑induced 1p‑Enh/*CYP4B1* regulatory axis in both HF and LC, suggesting that *CYP4B1* may serve as a potential therapeutic target for the concurrent treatment of HF and LC.

## Introduction

HF and LC are two major global health challenges that significantly contribute to morbidity and mortality [1,2]. Epidemiological studies have highlighted a frequent coexistence of these two conditions [3–5]. A retrospective analysis of U.S. death‑certificate data identified 214,785 deaths among adults aged ≥45 years with concomitant LC and chronic ischemic heart disease, corresponding to an age‑adjusted mortality rate of 8.4 per 100,000 [6]. In a German administrative‑claims cohort of 70,698 incident LC cases (2005–2019), 16.5% of patients had pre‑existing congestive heart failure (CHF), and these comorbid individuals exhibited significantly poorer survival compared to LC patients without CHF [7]. Moreover, El‑Rayes et al. reported that among all cancer types, lung cancer patients have the highest prevalence of pre‑existing cardiovascular disease, including a high burden of HF, and the greatest incidence of cardiovascular events following diagnosis. These observations are driven by shared risk factors such as smoking and overlapping pathophysiological mechanisms [8]. Despite this substantial comorbidity and its adverse impact on survival and healthcare utilization, the shared molecular drivers of HF–LC coexistence remain poorly defined.

Chronic cigarette smoking is a major environmental factor contributing to both HF and LC. Tobacco combustion generates high levels of reactive oxygen species (ROS), causing endothelial dysfunction, lipid peroxidation, and DNA damage in cardiac and pulmonary tissues [9]. In the heart, these insults drive myocardial remodeling and fibrosis [10], while in the lung, they initiate carcinogenic processes. Moreover, smoking disrupts immune surveillance by directing macrophage polarization toward a tumor‑promoting M2 phenotype, exacerbating cardiac dysfunction and facilitating malignant progression [11,12]. However, the molecular pathways by which smoking drives HF–LC comorbidity still need to be elucidated.

*CYP4B1* is one of the mammalian cytochrome P450 monooxygenases (CYP450), which plays a key role at the interface of endogenous and xenobiotic metabolism [13,14]. From a pathological perspective, *CYP4B1* is believed to be involved in several types of cancer due to altered gene expression levels in tumor tissues compared to normal tissues [15]. Lin et al. demonstrated that *CYP4B1* expression is associated with advanced tumor stage, lymph node metastasis, higher histological grade, vascular and perineural invasion, and increased mitotic activity in upper tract and bladder urothelial carcinomas [16]. In the Chinese Han population, missense mutations in *CYP4B1* have been shown to increase susceptibility to LC [17]. Although clinical studies have linked *CYP4B1* expression to LC, its role in HF remains largely uncharacterized. Currently, Han et al. (2025) are the only investigators demonstrating that thyroid‑stimulating hormone (TSH) promotes cardiomyocyte hypertrophy by modulating *CYP4B1* expression [18]. Transcriptionally, *CYP4B1* expression is controlled by a ligand–receptor interaction that induces heterodimer formation, promotes rapid nuclear translocation, and facilitates promoter binding, thereby enhancing its transcriptional activity [19]. Previous studies have identified nuclear receptor–binding motifs within the *CYP4B1* promoter, and sequence analysis revealed consensus hypoxia‑responsive elements—including binding sites for HIF‑1α, nuclear factor‑κB (NF‑κB), and activator protein‑1 (AP‑1) [15]. Functional assays demonstrated that HIF‑1 was rapidly activated under low-oxygen conditions and specifically bound the *CYP4B1* promoter, resulting in transcriptional upregulation of *CYP4B1* expression [20]. Nevertheless, the precise molecular mechanisms by which *CYP4B1* drives HF–LC comorbidity remain poorly understood, limiting the development of effective therapeutic strategies.

**Fig 1:**
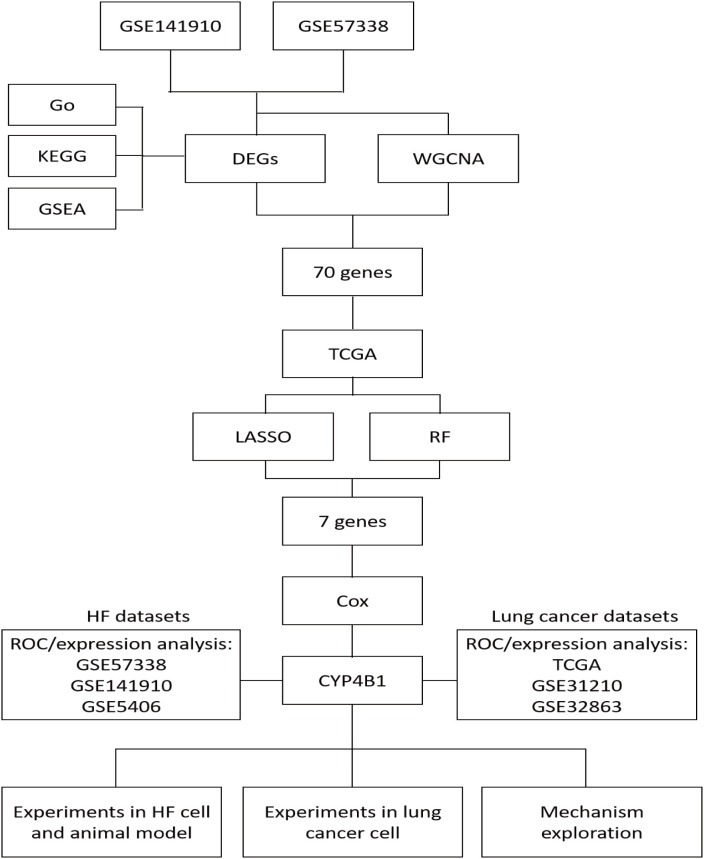
Flow chart of the study process.

Given the significant clinical burden of concurrent HF and LC, and the lack of clearly defined molecular connections, we employed integrative bioinformatics to identify *CYP4B1* as a key comorbidity gene (Fig 1). Functional assays demonstrated that *CYP4B1* suppresses lung cancer progression and mitigates pathological cardiac remodeling. Mechanistically, we identified a distal enhancer, 1p‑Enh, as a critical transcriptional regulator of *CYP4B1*. Furthermore, we demonstrated that the tobacco carcinogen NNK perturbs 1p‑Enh activity, thus linking smoking exposure to dysregulated *CYP4B1* expression and HF–LC comorbidity. Collectively, these findings establish the NNK‑induced 1p‑Enh/*CYP4B1* axis as a promising therapeutic target for the concurrent treatment of HF and LC.

## Materials and methods

### Data sources

The GSE57338 cohort comprised 313 samples, including 177 patients with HF and 136 non-HF controls, data were generated on the Affymetrix Human Gene 1.1 ST Array (GPL11532). The GSE141910 cohort included 366 samples, of which 200 were HF patients and 166 were non-HF controls, sequencing was performed on the Illumina HiSeq 2500 platform (GPL16791). The TCGA‑LUAD cohort retrieved via the UCSC Xena Browser consisted of 585 specimens, including 526 lung adenocarcinoma tissues and 59 adjacent normal tissues (https://xenabrowser.net/). DEGs between disease and control groups was conducted using the limma package in R [21], with significance thresholds set at *p* < 0.05 and |log_2_ fold change| > 0.5. Volcano plots were constructed to visualize the distribution of DEGs.

For external validation, three independent GEO datasets were employed. The GSE5406 cohort comprised 210 samples (194 HF patients and 16 non-HF controls; GPL96, Affymetrix Human Genome U133A Array). The GSE31210 cohort included 246 samples (226 lung adenocarcinoma tissues and 20 normal lung tissues; GPL570, Affymetrix Human Genome U133 Plus 2.0 Array). The GSE32863 cohort consisted of 116 samples, evenly split between 58 lung adenocarcinoma tissues and 58 adjacent normal tissues, generated on the Illumina HumanWG‑6 v3.0 expression bead chip (GPL6884).

### Functional and pathway enrichment analyses

Functional enrichment analyses of DEGs were performed using the clusterProfiler package in R [22], focusing on the Kyoto Encyclopedia of Genes and Genomes (KEGG) and Gene Ontology (GO) resources. GO analysis groups DEGs into three domains: molecular function (MF), cellular component (CC), and biological process (BP), thereby offering insights into the biological roles of DEGs. KEGG analysis reveals potential signaling pathways relevant to HF and LC pathogenesis.

### Weighted gene co-expression network analysis (WGCNA)

WGCNA was applied to construct co-expression networks for the GSE57338 and GSE141910 datasets, following the scale-free topology criterion [23]. The pick Soft Threshold function in the WGCNA package was employed to determine the soft threshold power and adjacency values. The adjacency matrix was transformed into a topological overlap matrix, and its dissimilarity was computed for hierarchical clustering analysis. Co-expressed gene modules were identified using the dynamic tree-cutting method. Pearson correlation coefficients between module eigengenes and HF were calculated to identify the hub module.

### Identification of the signature gene

Candidate hub genes were identified through the intersection of DEGs and key module genes. The least absolute shrinkage and selection operator (LASSO) [24] and Random Forest algorithms [25] were applied to identify hub genes associated with LC. Cox regression analysis was performed using the coxph() function from the survival package in R, with the proportional hazards assumption evaluated at a significance threshold of 0.05. The diagnostic performance of each selected signature gene was assessed using the area under the receiver operating characteristic (AUC-ROC) curve, with an AUC-ROC value>0.7 considered indicative of favorable diagnostic performance.

### Immune cell infiltration analysis

Immune cell infiltration was assessed using the CIBERSORT algorithm with the LM22 signature matrix. Gene expression datasets for HF (GSE141910 and GSE57338) and LC (TCGA) were preprocessed, with batch effects corrected using the “ComBat” function. Samples were stratified into CYP4B1 high- and low-expression groups based on median expression levels. The proportions of 22 immune cell types were estimated, and group differences were assessed using the Wilcoxon rank-sum test. Analyses were performed in R (version 4.3.1).

### Reagents and Antibodies

DMEM, fetal bovine serum (FBS), RPMI1640 medium, and DMEM/F-12 were purchased from Gibco (MA, USA). Penicillin & streptomycin, crystal violet, soft agar, MTT, 4% paraformaldehyde, proteinase K, and Phalloidin-Tetramethylrhodamine B isothiocyanate were obtained from Sigma (MO, USA). Trizol reagent was sourced from Invitrogen. Angiotensin II (Ang Ⅱ) was purchased from MCE (Princeton, USA). *EcoRI* and T4 ligase were obtained from TAKARA (Kusatsu, Japan). Antibodies against STAT3 and p-STAT3 were purchased from Cell Signaling Technology (Boston, USA). The antibody against GAPDH was purchased from Proteintech (Chicago, USA).

### Cell culture

The A549 lung cancer cell line was obtained from the American Type Culture Collection and cultured in DMEM supplemented with 10% FBS, 1% penicillin and streptomycin. H1703 lung cancer cell line was kindly supplied by Prof. Yonggang Zhao (Suzhou Institute of Systems Medicine) and cultured in RPMI1640 medium supplemented with 10% FBS, 1% penicillin and streptomycin. Beas-2B normal bronchial epithelial cell line was kindly supplied by Prof. Chaojun Li (Nanjing University) and cultured in DMEM supplemented with 10% FBS, 1% penicillin and streptomycin. The AC-16 cardiomyocyte cell line was obtained from the Stem Cell Bank of the Chinese Academy of Sciences and maintained in DMEM/F-12 with 10% FBS, 1% penicillin and streptomycin. All the cells were cultured at 37°C in a humidified atmosphere containing 5% CO_2_.

### Quantitative reverse transcription–polymerase chain reaction (qRT-PCR)

Total RNA was extracted using Trizol reagent following the manufacturer’s protocol. Total RNA (500 ng) was reverse‐transcribed using the Transcriptor First Strand cDNA Synthesis Kit (Roche) according to the manufacturer’s instructions. Briefly, RNA was mixed with Random Hexamer Primer and dNTP mix, incubated at 65 °C for 10 min, then chilled on ice. Reverse transcription was performed with Transcriptor Reverse Transcriptase and buffer at 50 °C for 30 min, followed by enzyme inactivation at 85 °C for 5 min. qRT-PCR was conducted using SYBR Green and the following thermal cycling conditions: initial denaturation at 95°C for 10 min, followed by 40 cycles of denaturation at 95°C for 15 s, annealing at 60°C for 15 s, and extension at 72°C for 15 s. The sequences of the primers used are listed in S6 Table.

### Western blotting

Whole-cell lysates were prepared from cells. Western blot was performed as described previously [26]. The protein samples were separated into 10% sodium dodecyl sulfate–polyacrylamide gel. The primary antibodies for STAT3 (1:1000), P-STAT3 (1:1000) and GAPDH (1:5000) were used. The protein bands were visualized by the Chemiluminescence gel imaging system.

### Laser confocal experiment

The cells were fixed with 4% paraformaldehyde for 20 min at room temperature, followed by 0.1% Triton X-100 PBS for 5 min. Blocking was performed with 1% bovine serum albumin (BSA) PBS for 20 min. The cells were incubated with the staining solution of Phalloidin-TetramethylrhodamineB isothiocyanate for 30 min at room temperature in the dark. After each step, the cells were washed three times with PBS. Confocal microscopy (Olympus BX63) was utilized to capture images, followed by the use of Image J to analyze the cell area.

### Mouse model of cardiac remodeling

Six-week-old BALB/c mice (Institute of Model Animals, Nanjing University) were randomly divided into two groups (n = 10 per group [5 per sex]). Ang Ⅱ was administered at 1000 ng/kg/min via an osmotic mini-pump (Alzet MODEL 1004, USA) for 4 weeks, following established protocols [19]. At the end of the treatment, the mice were euthanized using sodium pentobarbital. The hearts were either snap-frozen in liquid nitrogen for subsequent qRT-PCR and western blot analyses or fixed in 4% paraformaldehyde in PBS for histological examination. Fixation was carried out at room temperature for 4 h.

### Cell migration and invasion assay, Colony formation and Soft agar assay

***Cell migration*** 1 × 10^5^ cells were seeded onto a fibronectin-coated polycarbonate membrane insert in a transwell apparatus (Corning, USA) with 100 µl of serum-free DMEM (for A549 cells) or RPMI1640 (for H1703 cells). The lower chamber was filled with 500 µl of DMEM or RPMI1640 containing 20% FBS (for A549 and H1703 cells, respectively). Then, the cells were incubated at 37°C in a humidified atmosphere with 5% CO_2_ for 36 h. Afterward, non-migrated cells on the upper surface of the membrane were removed by washing with PBS, and the migrated cells were stained with crystal violet. Cell counts were performed under a 20 × objective (fixed field of view) using a Zeiss Axio Observer microscope equipped with a 20 × objective lens. Five fields per insert were imaged in a systematic pattern (left‑to‑right, top‑to‑bottom) to ensure consistent sampling. All counts were conducted in a blinded manner to avoid bias.

***Cell invasion*** Cell invasion was assessed using a matrigel coated transwell chamber (BD Biosciences). Matrigel was diluted to 3 mg/ml in serum-free DMEM (for A549 cells) or RPMI1640 (for H1703 cells) and applied to the membrane surface. Following transient transfection, 5 × 10^4^ cells suspended in 200 µl DMEM or RPMI1640 containing 10% FBS (for A549 and H1703 cells, respectively) were seeded onto the matrigel coated membrane, and 500 µl DMEM or RPMI1640 containing 20% FBS (for A549 and H1703 cells, respectively) was added to the lower chamber. After 24–48 h culture, the matrigel layer was carefully removed, and the migrated cells were stained and quantified under a microscope.

***Colony formation assay*** A total of 300 transfected cells with CYP4B1 plasmid were seeded into each well of a six-well plate containing 1 ml medium supplemented with 10% FBS. DMEM was used for A549 cells, and RPMI1640 for H1703 cells. The culture medium was replaced every 3 days and removed after 14 days. To assess cell proliferation, the cells were fixed with 4% paraformaldehyde and then stained and quantified under a microscope.

**Soft agar assay** A total of 2.5 × 10³ NNK-induced Beas-2B cells were seeded in 2 mL DMEM containing 0.2% soft agar. After 3–4 weeks of culture, colonies in the soft agar were stained with 0.05% MTT at 37°C for 5h and then counted under a microscope [27].

### NNK treatment assays

AC‑16 cardiomyocytes and Beas-2B cells were treated with NNK. For short‑term exposure, cells were incubated with 10 μM NNK for 24 hours. For long‑term exposure, cells were continuously treated with 450 μM NNK for 15 consecutive passages.

### Luciferase reporter assay

Beas-2B, H1703, A549, and AC-16 cells were seeded in 24-well plates at a density of 0.5 × 10^5^ cells per well and cultured until they reached 80% confluence. Two candidate enhancer fragments were PCR‑amplified from human genomic DNA and directionally cloned into the *KpnI* and *XhoI* sites of the pGL3‑promoter vector. Enhancer 1 (Enh1; hg38: chr1:46819643–46821973) was amplified using the forward primer 5′‑CGGGGTACCGGAAGGGAAGGAACCTGTAAGA‑3′ (KpnI site underlined) and the reverse primer 5′‑CCGCTCGAGTTGCGATAGTTTGCTGAGAATG‑3′ (XhoI site underlined). Enhancer 2 (1p‑Enh; hg38: chr1:46822127–46824170) was amplified using the forward primer 5′‑CGGGGTACCAAATGACGAGTTAATGGGTGCA‑3′ and the reverse primer 5′‑CCGCTCGAGTAGACACGGGTCAGGAATGGTA‑3′. All constructs were sequence‑verified before use.

For the reporter assays, each well was then transfected with 500 ng vector DNA and 0.5ng pRL-SV40 (Promega) by using Lipofectamine 3000 (Invitrogen). Cells transfected with the empty pGL3-promoter vector served as negative control. After 48h culture, luciferase activity was measured using the Dual-Luciferase® Reporter Assay System (Promega). The luciferase signal was normalized by using Renilla luciferase values as an internal control.

### Chromosome-conformation capture (3C) assay

The 3C experiment was conducted in accordance with the methodology described in previously published literature [28]. Briefly, approximately 1 × 10^6^ Beas-2B or AC-16 cells were crosslinked using formaldehyde at room temperature, quenched with glycine, and then lysed to isolate their cell nuclei. Their cell nuclei were digested with *EcoRI* at 37°C, and their enzymes were inactivated. The samples were then diluted in 700 μl of ligation mixture containing 100 U T4 ligase and incubated overnight at 16°C. Ligated chromatin was treated with proteinase K and purified using phenol-chloroform extraction. Specific primers were used to analyze interactions between *CYP4B1* and 1p-Enh, and the results were confirmed via DNA sequencing. The primer sequences are provided in S7 Table.

### Ethical statement

All the animal procedures adhered to the 3Rs principles of animal welfare and were approved by the Laboratory Animal Welfare Ethics Committee of Nanjing Medical University (Permit Number: IACUC-2304049). All the animal husbandry and experiments were conducted in strict accordance with regulations governing the management and use of laboratory animals.

### Statistical analysis

Unless otherwise specified, all experiments were performed with at three independent biological replicates; luciferase reporter assays were conducted in quadruplicate. Data are presented as mean ± SD. Comparisons between two groups were made using the unpaired Student’s t‑test, and p < 0.05 was considered statistically significant.

## Results

### DEGs in HF patients and functional enrichment analysis

**Fig 2:**
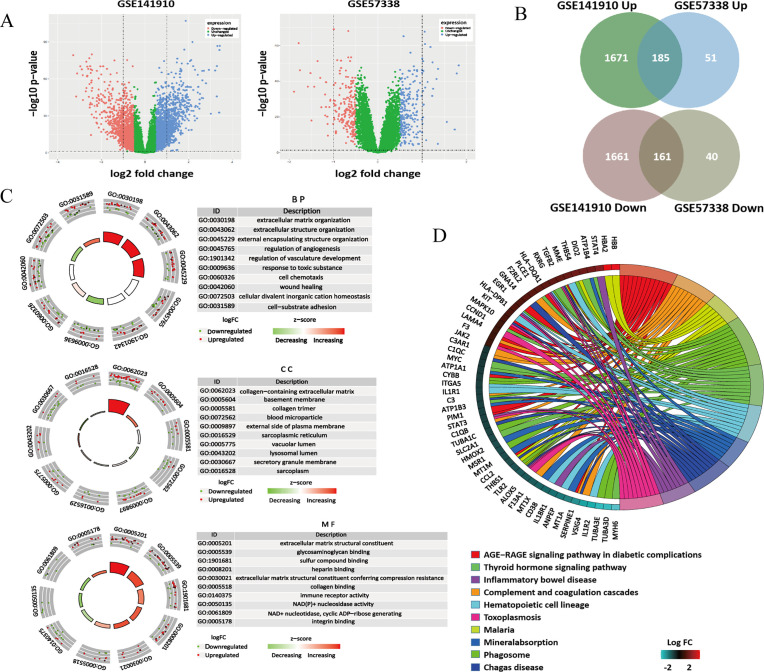
DEGs in HF and cluster analysis. **(A)** Volcano plot showing the distribution of DEGs in HF samples (adjusted p < 0.05 and |logFC| > 0.5). Each point represents a gene. Blue, red, and green dots indicate significantly upregulated genes, significantly downregulated genes, and genes with non-significant changes, respectively. **(B)** Venn diagram illustrating the intersection of the DEGs between the GSE141910 and GSE57338 datasets, identifying 346 overlapping genes (185 upregulated and 161 downregulated). **(C)** GO enrichment analysis showing the top BPs, CCs, and MFs associated with the overlapping DEGs. **(D)** KEGG pathway enrichment analysis highlighting key biological pathways involved in HF pathogenesis.

In this study, we identified DEGs between HF patients and healthy controls using the GSE141910 and GSE57338 datasets. Utilizing the “limma” package in R, we detected 346 DEGs, comprising 185 upregulated and 161 downregulated genes. The volcano plot of these DEGs is shown in Fig 2A, and the Venn diagram of the intersection of DEGs from the two datasets is shown in Fig 2B. Functional enrichment analysis revealed significant BPs related to ECM organization (p = 9.65E-11), supported by enrichment in collagen-containing ECM (cellular component, p = 2.12E-29) and ECM structural constituents (molecular function) (Fig 2C, S1 Table). KEGG pathway analysis highlighted key pathways, including the AGE-RAGE signaling pathway in diabetic complications (p = 1.37E-06), complement and coagulation cascades (p = 8.89E-05), and phagosome formation (p = 0.0001) (Fig 2D, S2 Table), all implicated in inflammatory responses and metabolic dysregulation central to HF pathogenesis. Additional pathways, such as thyroid hormone signaling (p = 0.0002) and mineral absorption (p = 0.0002), suggested further regulatory mechanisms in disease progression. GSEA further elucidated HF’s molecular landscape, identifying pathways including collagen-containing ECM (GO_GSEA), viral mRNA translation (Reactome_GSEA), and ribosome pathway (KEGG_GSEA) (S1 Fig, S3 Table). These findings collectively underscore the critical roles of ECM remodeling, inflammation, and metabolic dysregulation in HF.

### Essential gene modules identified in the weighted gene co-expression network of HF patients

**Fig 3:**
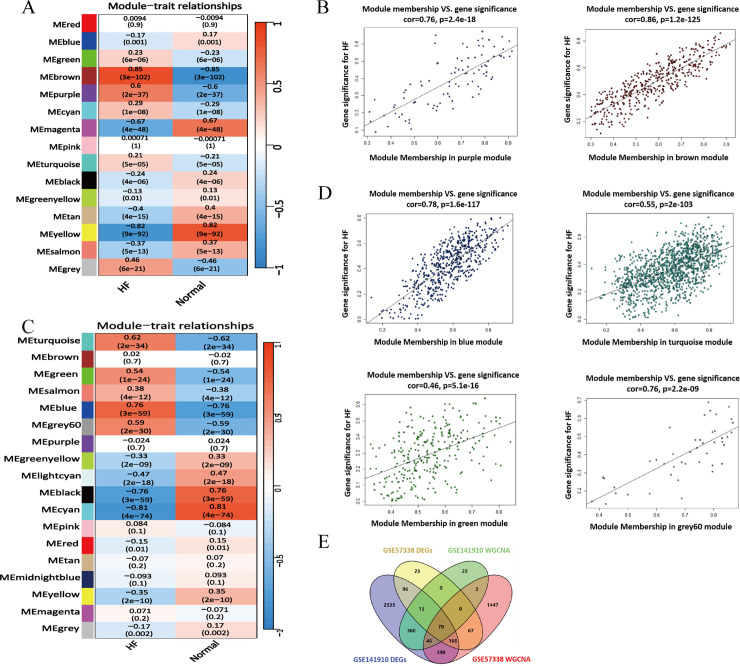
Identification of key gene modules associated with HF. **(A)** Heatmap illustrating the correlation between module eigengenes and HF/healthy phenotypes in the GSE141910 dataset. The numbers in each block represent the correlation coefficient and the corresponding p-value (in parentheses), with red and blue colors indicating positive and negative correlations, respectively. **(B)** Scatter plots demonstrating the correlation between module membership and gene significance within the purple and brown modules identified in GSE141910, both significantly associated with HF (cor = 0.76, 0.86; p < 0.001). **(C)** Heatmap depicting module-trait correlations for HF/healthy phenotypes in the GSE57338 dataset. **(D)** Scatter plots illustrating the correlation between module membership and gene significance within the blue, turquoise, green, and grey60 modules from GSE57338. High module membership indicates high importance of these genes within their respective modules. **(E)** Venn diagram showing the overlap of DEGs and key WGCNA modules from both datasets (GSE141910 and GSE57338). A total of 70 overlapping genes were identified, representing candidate hub genes potentially implicated in HF pathogenesis.

Next, we employed WGCNA to identify key gene modules associated with HF. Using the GSE57338 and GSE141910 datasets, we constructed co-expression networks with soft threshold powers of 2 and 5, respectively, achieving a scale-free topology fitting index (R²) of 0.9 and near-zero average connectivity, confirming network suitability (S2–S3 Fig). In the GSE141910 dataset, two modules (brown and purple) were identified (Fig 3A–3B), while four modules (blue, green, grey60, and turquoise) were identified in the GSE57338 dataset (Fig 3C–3D). Modules with correlation coefficients (r)>0.5 were selected for further analysis. By intersecting genes from these six modules with the previously identified DEGs, we identified 70 candidate genes potentially implicated in HF pathogenesis (Fig 3E, S4 Table). These genes are potential targets for functional validation.

### Identification of LC-related signature genes

**Fig 4:**
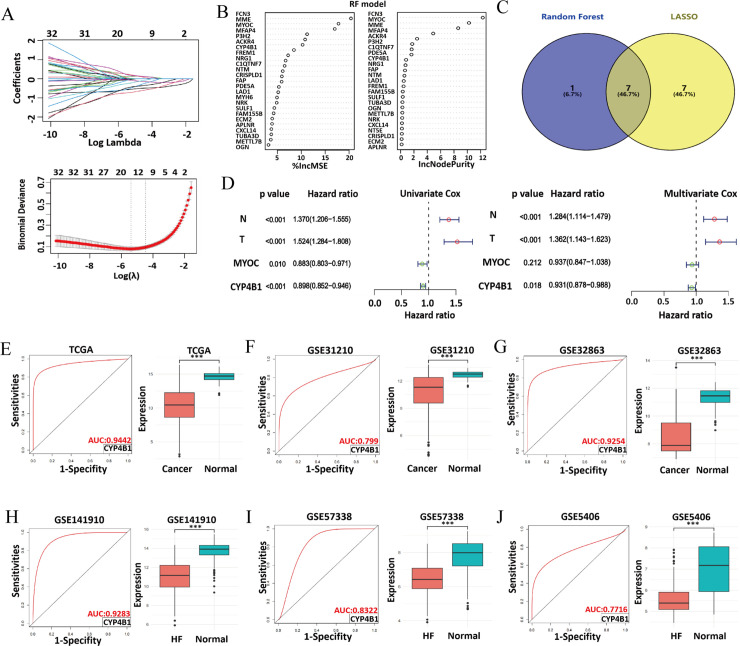
Screening for signature genes related to LC and HF. **(A)** LASSO regression analysis. The upper panel displays the coefficient profiles of the features as the penalty parameter (lambda) increases. The lower panel shows the selection of the optimal lambda using cross validation, where the error bars represent standard errors. **(B)** Variable importance plots from the RF model, ranking genes based on their predictive importance obtained using %IncMSE and IncNodePurity metrics. **(C)** Venn diagram illustrating the overlap of signature genes (7 in total) identified via LASSO and RF models. **(D)** Univariate and multivariate Cox regression analyses of the overlapping genes to evaluate their prognostic significance, with the hazard ratios indicated. Notably, CYP4B1 displays a high potential as a prognostic marker. **(E–J)** Diagnostic efficacy and expression levels of CYP4B1 in various datasets. Panels E, F, and G show results from cancer datasets (TCGA, GSE31210, and GSE32863, respectively), and panels H, I, and J show results from HF datasets (GSE141910, GSE57338, and GSE5406, respectively). ROC curves (left) and box plots of expression levels (right) highlight the diagnostic potential of CYP4B1 in distinguishing diseased samples from control samples, with the AUC values noted for each dataset.

To identify LC–associated signature genes from our 70 HF‑linked candidates, we employed two machine-learning algorithms using TCGA datasets. First, LASSO analysis identified 14 candidate genes (S5 Table, Fig 4A). Next, Random Forest analysis identified 8 signature genes by intersecting the top 10 genes ranked by IncMSE with the top 10 ranked by Node Purity (Fig 4B). Combining the results from both analyses led to the identification of *MYOC*, *P3H2*, *ACKR4*, *FCN3*, *CYP4B1*, *NRG1*, and *MME* as key signature genes (Fig 4C). We next subjected these seven genes to univariate Cox regression, which identified *MYOC* and *CYP4B1* as significantly prognostic. In multivariate Cox analysis, only *CYP4B1* remained an independent predictor of overall survival in LC (Fig 4D).

### *CYP4B1* is a common diagnostic biomarker of HF and LC

To evaluate *CYP4B1* as a diagnostic biomarker for HF and LC, we first analyzed its diagnostic performance using the TCGA database. CYP4B1 exhibited strong diagnostic performance for LC, with an AUC-ROC of 0.9442, and its expression was significantly downregulated in LC tissues compared to non-tumorous controls (Fig 4E). To further validate these findings, we assessed the diagnostic accuracy of CYP4B1 in two independent GEO cohorts. Consistent with the TCGA data, the AUC-ROC values were 0.799 in GSE31210 and 0.9254 in GSE32863, with reduced CYP4B1 expression observed in both datasets (Fig 4F-4G). Additionally, *CYP4B1* showed strong potential as a diagnostic biomarker for HF. AUC-ROC values were 0.9283 in GSE141910, 0.8322 in GSE57338, and 0.7716 in GSE5406, with its expression consistently downregulated across all HF datasets (Fig 4H-4J). These results indicated *CYP4B1* as a promising diagnostic biomarker for both HF and LC.

### CYP4B1 correlates with immune cell infiltration in HF and LC

**Fig 5:**
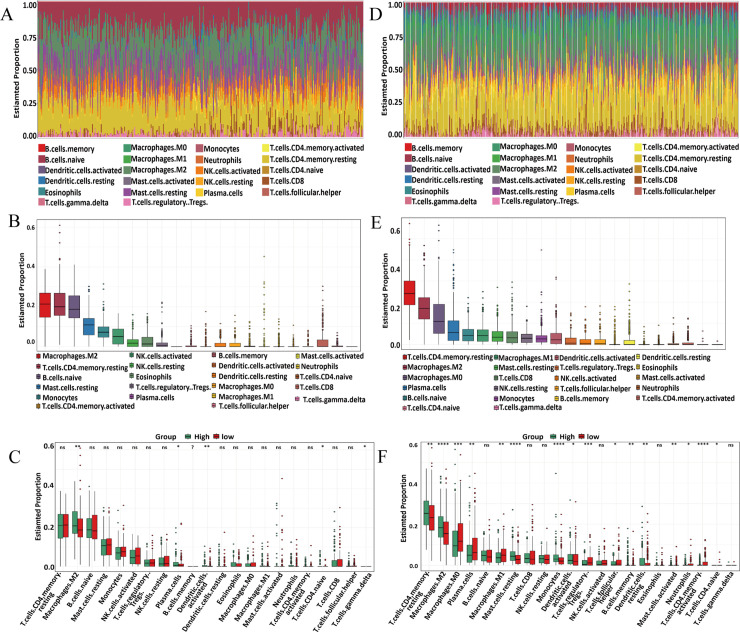
CYP4B1-associated immune-cell infiltration in HF and LC samples. **(A)** Immune cell infiltration profiles of 22 immune cell types in HF samples were determined using the CIBERSORT algorithm. **(B)** The top three infiltrating immune cell types in HF samples were M2 macrophages, resting memory CD4^+^ T cells, and naive B cells. **(C)** Comparison of immune-cell infiltration between *CYP4B1* high-expression and low-expression groups in HF samples. Among the 22 immune cell types analyzed, infiltration of M2 macrophages was significantly higher in the *CYP4B1* high-expression group, alongside differences in the infiltration levels of other immune cell types. **(D)** Immune cell infiltration profiles of 22 immune cell types in LC samples from TCGA. **(E)** The top three infiltrating immune cell types in LC samples were resting memory CD4^+^ T cells, M2 macrophages, and M0 macrophages. **(F)** Comparison of immune cell infiltration between *CYP4B1* high-expression and low-expression groups in lung cancer samples. Resting memory CD4^+^ T cells and M2 macrophages showed higher infiltration while M0 macrophages showed lower infiltration in the *CYP4B1* high-expression group.

To explore the relationship between *CYP4B1* expression and immune cell infiltration, we first analyzed immune-cell proportions in HF samples combined from the GSE141910 and GSE57338 datasets. Batch effects were removed before analysis, and the samples were stratified into *CYP4B1* high-expression and low-expression groups based on the median *CYP4B1* expression level. Using the CIBERSORT algorithm, we assessed the infiltration of 22 immune cell types. The top three infiltrating immune cells were M2 macrophages, resting memory CD4 + T cells, and naive B cells (Fig 5A-5B). Notably, infiltration of M2 macrophages was significantly higher in the *CYP4B1* high-expression group than in the low-expression group (Fig 5C). A similar analysis was performed on LC tissues from TCGA. The top three infiltrating immune cells in the LC samples were resting memory CD4 + T cells, M2 macrophages, and M0 macrophages (Fig 5D-5E). Importantly, infiltration of resting memory CD4 + T cells and M2 macrophages was significantly higher but infiltration of M0 macrophages was significantly lower in the *CYP4B1* high-expression group than in the low-expression group (Fig 5F). These findings suggest *CYP4B1* modulates immune cell infiltration in both HF and LC.

### CYP4B1 inhibited Ang Ⅱ-induced cardiac hypertrophy and suppressed LC cell invasion, migration, and proliferation

**Fig 6:**
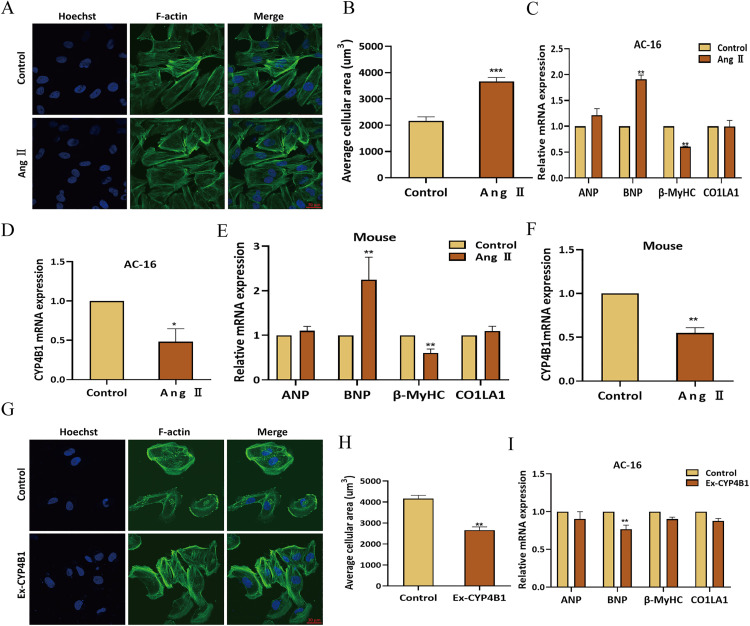
CYP4B1 overexpression protects AC-16 cells from Ang Ⅱ-induced hypertrophy. **(A)** Phalloidin staining (green) shows F-Actin reorganization in AC-16 cells treated with 10μM Ang **Ⅱ. (B)** Statistical analysis of Phalloidin staining indicated a significant increase in the average cell-surface area in Ang Ⅱ-treated AC-16 cells versus controls. **(C)** qRT-PCR analyses showed significant BNP upregulation in Ang Ⅱ-treated AC-16 cells. (**D**) qRT-PCR analyses showed significant *CYP4B1* decrease in Ang Ⅱ-treated AC-16 cells. **(E)** qRT-PCR analyses showed significant BNP upregulation in Ang Ⅱ-treated mouse cardiomyocytes. (**F**) qRT-PCR analyses showed significant *CYP4B1* decrease in Ang Ⅱ-treated mouse cardiomyocytes. **(G)** Phalloidin staining (green) shows F-Actin reorganization in AC-16 cells transfected with *CYP4B1*. **(H)** The average cell surface area is significantly decreased in CYP4B1-transfected AC-16 cells versus untransfected cells (n = 3 per group). **(I)** qRT-PCR results showed BNP downregulation in CYP4B1-transfected AC-16 cells versus untransfected cells (n = 3 per group). ^*^p < 0.05, ^**^p < 0.01, ^***^p < 0.001.

We explored the role of *CYP4B1* in HF by evaluating hypertrophic responses in AC-16 cells exposed to Ang II. Ang Ⅱ treatment induced F-actin stress fiber formation and increased cell surface area by 1.8-fold (Fig 6A–6B). Among hypertrophy-related genes (*ANP*, *BNP*, *β-MyHC*, *COL1A1*), only *BNP* mRNA levels significantly increased (1.9-fold; p < 0.01), while *CYP4B1* expression was suppressed (Fig 6C–6D), suggesting its inhibitory role in cardiac hypertrophy. In vivo, Ang Ⅱ-treated mice exhibited *BNP* upregulation and *CYP4B1* downregulation, consistent with in vitro findings (Fig 6E–6F). Overexpression of *CYP4B1* in AC-16 cells attenuated Ang Ⅱ-induced increases in cell surface area and *BNP* expression (Fig 6G-6I), highlighting its protective role.

**Fig 7:**
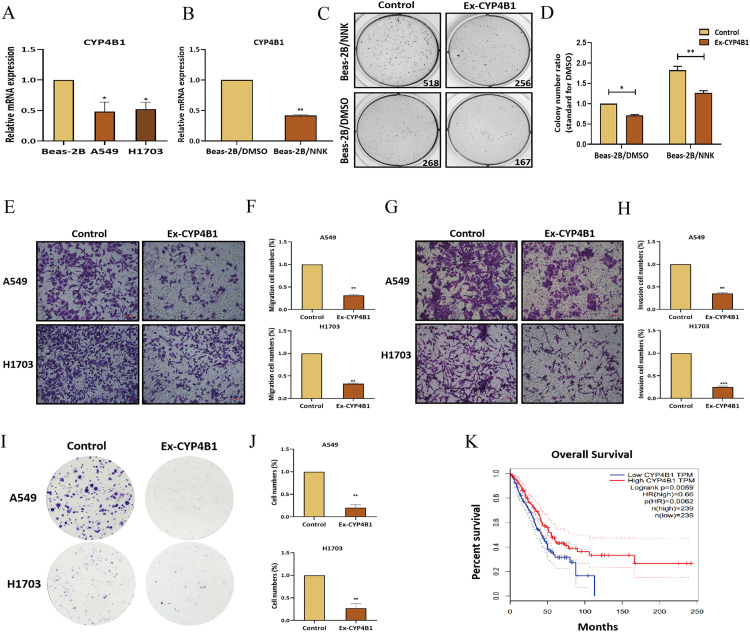
CYP4B1 inhibits the migration, invasion, and proliferation of LC cells in vitro. **(A)** qRT-PCR analyses showed significant *CYP4B1* downregulation in LC cell lines A549 and H1703, compared with Beas-2B cells (n = 3 per group). **(B)** qRT-PCR showed significant *CYP4B1* downregulation in Beas-2B cells treated with NNK for 15 generations (n = 3 per group). **(C)** Soft agar assays showed the effect of *CYP4B1* on NNK-induced malignant transformation of Beas-2B cells. **(D)** Statistical analysis of Soft-agar assays showed that *CYP4B1* overexpression suppressed NNK-induced malignant transformation of Beas-2B cells (n = 3 per group). **(E)** Transwell migration assays showed the migration ability of A549 and H1703 cells transfected with *CYP4B1*. **(F)** Statistical analysis of transwell migration assays showed that *CYP4B1* overexpression significantly suppressed the migration of A549 and H1703 cells. **(G)** Transwell invasion assays were performed to evaluate the effect of *CYP4B1* overexpression on the invasive ability of A549 and H1703 cells. **(H)** Statistical analysis of transwell invasion assays showed that *CYP4B1* overexpression significantly reduced the invasion of A549 and H1703 cells. **(I)** Colony forming assays were performed to determine the effect of *CYP4B1* overexpression on the proliferation of A549 and H1703 cells. **(J)** Statistical analysis of Colony forming assays showed that *CYP4B1* overexpression significantly repressed the proliferation of A549 and H1703 cells. **(K)** Kaplan–Meier analysis revealed a positive correlation between *CYP4B1* expression and the survival of LC patients (http://gepia.cancer-pku.cn). ^*^p < 0.05, ^**^p < 0.01, ^***^p < 0.001.

In LC, *CYP4B1* was significantly downregulated in A549 and H1703 cells compared to bronchial epithelial cells (Beas-2B) and further reduced in NNK-treated Beas-2B cells (Fig 7A–7B). *CYP4B1* overexpression inhibited NNK-induced malignant transformation, as evidenced by reduced colony formation (Fig 7C–7D). In A549 and H1703 cells, *CYP4B1* overexpression significantly reduced both cell migration, invasion and proliferation (Fig 7E–7J). Survival analysis further indicated that LC patients with high *CYP4B1* expression had significantly better prognoses than those with low expression (p < 0.01, Fig 7K).

### CYP4B1 suppresses HF and LC progression via STAT3 pathway

**Fig 8:**
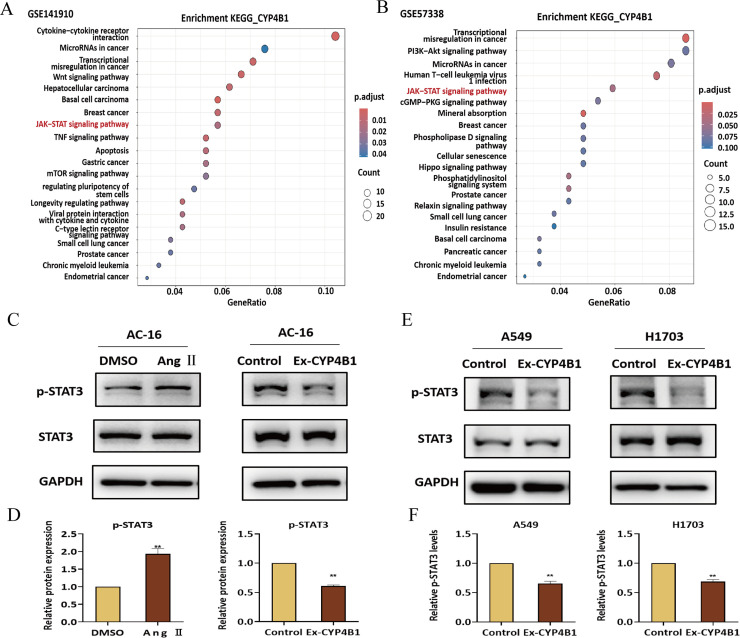
CYP4B1 inhibits the STAT pathway in cardiomyocytes and LC cells. **(A** and **B)** KEGG cluster analysis of genes significantly correlated with *CYP4B1* expression. **(C)** WB was performed to evaluate the effect of Ang Ⅱ treatment and *CYP4B1* overexpression on STAT3 phosphorylation in AC-16 cells. **(D)** Statistical analysis WB showed that Ang Ⅱ treatment significantly increased STAT3 phosphorylation in AC-16 cells, whereas *CYP4B1* overexpression inhibited Ang Ⅱ–induced STAT3 phosphorylation. **(E)** WB was performed to evaluate the effect of *CYP4B1* overexpression on STAT3 phosphorylation in A549 and H1703 cells. **(F)** Statistical analysis of WB showed that *CYP4B1* overexpression reduced STAT3 phosphorylation in both A549 and H1703 cells. ^**^P < 0.01.

To elucidate the mechanism whereby *CYP4B1* exerts its inhibitory effects in HF and LC, we analyzed the genes that are highly correlated with *CYP4B1* in two HF datasets (GSE141910 and GSE57338). KEGG enrichment analysis revealed significant enrichment of the JAK-STAT signaling pathway in both gene sets (Fig 8A–8B), suggesting that *CYP4B1* exerts its biological effects through this pathway. Functional validation in Ang Ⅱ-treated AC-16 cardiomyocytes showed that Ang Ⅱ upregulated STAT3 phosphorylation (p-STAT3), which was attenuated by *CYP4B1* overexpression (Fig 8C–8D). Furthermore, the expression levels of several STAT3 downstream genes, including TGFβ1 and SOCS3, were also significantly reduced in CYP4B1-overexpressing AC-16 cells (S4A-5B Fig). Similarly, in A549 and H1703 LC cells, *CYP4B1* overexpression significantly downregulated p-STAT3 (Fig 8E–8F), and this was accompanied by a marked decrease in the expression of STAT3 target genes such as TGFβ1 and CCND1(S4C-4D Fig). These findings suggest that *CYP4B1* exerts inhibitory effects in HF and LC, at least in part, through modulation of STAT3 pathway.

### CYP4B1 transcription is regulated by 1p-Enh through chromatin looping

**Fig 9:**
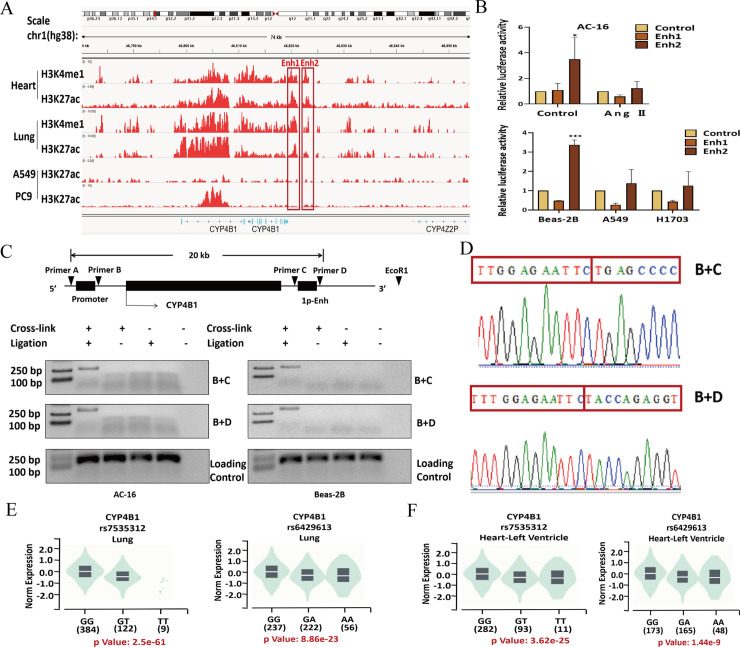
Identification of a novel enhancer regulating CYP4B1 expression. **(A)** Overview of the H3K4me1 and H3K27ac modifications near the 3′ UTR region of *CYP4B1* in the heart and lung tissues, as well as in A549 and PC9 LC cells. Two enhancer candidate regions, Enh1 and Enh2, were identified. **(B)** Luciferase reporter assays showed Enh2 exhibited significantly higher enhancer activity in AC-16 and Beas-2B cells, whereas Enh1 showed no enhancer activity in any of the tested cell lines (n = 3 per group). **(C)** Schematic diagram showed the relative positions of the CYP4B1 promoter, 1p-Enh enhancer, *EcoRI* restriction enzyme cut sites, and PCR primers for 3C assays (upper panel). 3C assays revealed a physical interaction between the *CYP4B1* promoter and 1p-Enh element in Beas-2B and AC-16 cells. **(D)** DNA sequencing confirmed that the PCR products resulted from the ligation of the 1p-Enh and *CYP4B1* promoter regions. **(E)** eQTL analysis revealed that rs7535312 and rs6429613 within 1p-Enh are associated with high *CYP4B1* expression in the lungs. **(F)** Results from eQTL analysis confirmed that 1p-Enh regulates *CYP4B1* expression in the heart. ^*^P < 0.05, ^**^P < 0.01, ^***^P < 0.001.

Alterations in myocardial DNA methylation play a pivotal role in the pathogenesis of HF [29]. To explore the mechanism whereby *CYP4B1* is downregulated in HF and LC, we first assessed the involvement of any DNA methylation in the CYP4B1 promoter. Bioinformatic analysis revealed no CpG island in the promoter region (S5 Fig), suggesting that DNA methylation of the promoter is unlikely to account for the *CYP4B1* downregulation in HF and LC. Recent evidence highlights inactivation of enhancers as a key factor in the pathogenesis of complex diseases. By interrogating ENCODE ChIP‑Seq tracks for H3K4me1 (a mark of poised enhancers) and H3K27ac (a mark of active enhancers), we identified two candidate enhancers (Enh1 and Enh2) near the CYP4B1 3′ UTR, characterized by both histone modifications (Fig 9A). Luciferase reporter assays showed that Enh2, but not Enh1, exhibited strong activity in AC-16 cardiomyocytes and Beas-2B cells, with no activity in H1703 and A549 LC cells or Ang Ⅱ-treated (long‑term exposure) AC-16 cells (Fig 9B). Given its chromosomal location at 1p34, we designated the active enhancer as 1p-Enh and focused subsequent analyses on its potential impact on *CYP4B1* expression. Enhancers typically regulate gene expression through chromatin looping, allowing distal enhancers to interact with the promoters of target genes. To investigate this interaction, we performed 3C assays in AC-16 and Beas-2B cells. Our results revealed chromatin looping between 1p‑Enh and the *CYP4B1* promoter in AC‑16 and Beas‑2B cells, whereas no looping signal was detected in negative controls (Fig 9C–9D). To further investigate the regulatory potential of 1p-Enh, we performed expression quantitative trait loci (eQTL) analysis by using the GTEx v8 database. Two germline variants, rs7535312 (G > T) and rs6429213 (G > A), were found in the 1p-Enh element. Carriers of the mutant alleles exhibited significantly lower *CYP4B1* expression than wild-type tissues in both lung and myocardial samples (Fig 9E–F). These data further confirm that 1p-Enh regulates *CYP4B1* expression.

### 1p-Enh activity increases with short-term NNK exposure but decreases with prolonged exposure

**Fig 10:**
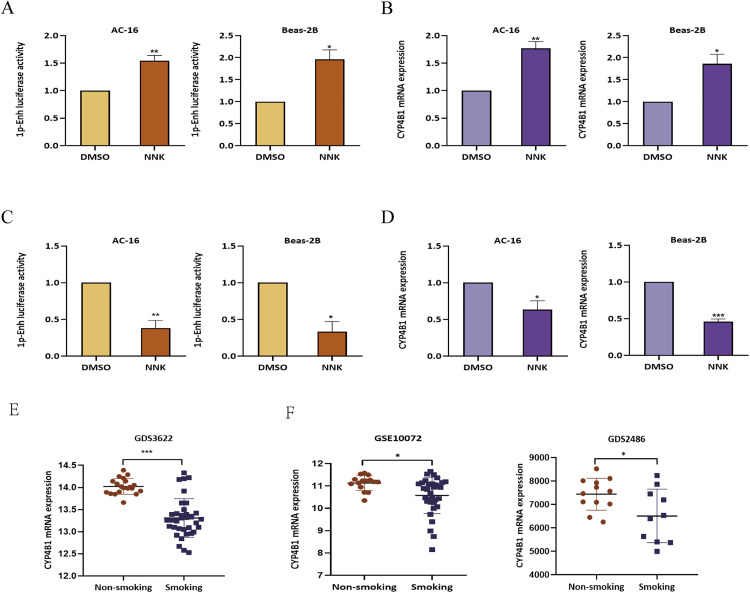
Effect of NNK on 1p-Enh activity and CYP4B1 expression. **(A)** Luciferase reporter assays showed a significant increase in 1p-Enh activity in AC-16 and Beas-2B cells following exposure to NNK for 24h. **(B)** qRT-PCR analyses showed significant *CYP4B1* upregulation in AC-16 and Beas-2B cells following exposure to NNK for 24h. **(C)** Luciferase reporter assays showed a significant decrease in 1p-Enh activity in AC-16 and Beas-2B cells following exposure to NNK for 15 generations. **(D)** qRT-PCR results showed significant *CYP4B1* downregulation in AC-16 and Beas-2B cells following exposure to NNK for 15 generations. **(E** and **F)** *CYP4B1* was significantly downregulated in mice exposed to cigarette smoke. ^*^P < 0.05, ^**^P < 0.01, ^***^P < 0.001.

Given the substantial contribution of smoking to the pathogenesis of HF and LC [27], we investigated whether NNK exposure modulated 1p-Enh activity and influenced HF and LC progression. In AC-16 cardiomyocytes and Beas-2B bronchial epithelial cells transfected with a 1p-Enh luciferase reporter plasmid, short-term NNK exposure (24h) significantly increased 1p-Enh activity and upregulated *CYP4B1* expression compared to DMSO-treated controls (Fig 10A–10B), suggesting a protective role of *CYP4B1* against NNK-induced damage. In contrast, prolonged NNK exposure (15 generations) reduced 1p-Enh activity more than 2 folds and downregulated *CYP4B1* expression in both cell types (Fig 10C–10D). Consistent with these findings, in vivo data (GDS3622) showed *CYP4B1* downregulation in lung tissues of cigarette smoke-exposed mice (Fig 10E) and significantly lower *CYP4B1* expression in smokers compared to non-smokers (Fig 10F). These results demonstrate that NNK exposure dynamically regulates 1p-Enh activity, with short-term activation and long-term suppression of *CYP4B1*, potentially contributing to HF and LC progression.

## Discussion

Here, we demonstrate that *CYP4B1* serves as a shared molecular mediator of HF and LC, emphasizing its critical role in these comorbid conditions. Mechanistically, *CYP4B1* mitigates pathological cardiac remodeling and suppresses tumorigenesis by inhibiting the STAT3 signaling cascade, with its transcriptional activity regulated by a novel distal enhancer, 1p‑Enh. Collectively, our findings establish *CYP4B1* as a potential therapeutic target and biomarker for the integrated management of HF and LC.

Aberrant expression of *CYP4B1* in various cancers has attracted increasing research attention. Previous studies have reported significantly decreased *CYP4B1* expression in LC tissues [15], identifying it as both a prognostic biomarker and a potential therapeutic target, especially in lung adenocarcinoma [30]. Consistent with these findings, our analysis of TCGA and GEO datasets revealed marked downregulation of *CYP4B1* in LC, which was further validated by qPCR and the low endogenous expression observed in A549 and H1703 cell lines. Functional assays, including cell invasion, migration, and colony formation, demonstrated that *CYP4B1* overexpression markedly suppressed the malignant behavior of A549 and H1703 cells, supporting its tumor-suppressive role in LC. Furthermore, exposure of bronchial epithelial Beas-2B cells to NNK, a tobacco-specific carcinogen, significantly reduced *CYP4B1* expression during malignant transformation. Moreover, ectopic expression of *CYP4B1* effectively counteracted NNK-induced malignant transformation in Beas-2B cells. Together, these findings provide robust experimental evidence supporting the tumor-suppressive function of *CYP4B1* in LC.

So far, the relationship between *CYP4B1* and HF remains inadequately characterized. Recently, Han et al. demonstrated that TSH upregulated *CYP4B1* through the PI3K/AKT/CREB pathway to promote cardiac hypertrophy [31]. Conversely, our bioinformatic analysis of GEO datasets GSE141910 and GSE57338 revealed significant downregulation of *CYP4B1* in HF patient samples. Experimental assays corroborated these findings, showing significant *CYP4B1* downregulation in Ang Ⅱ-induced AC-16 hypertrophy in vitro and myocardial tissues from HF mouse models. Furthermore, overexpression of *CYP4B1* attenuated Ang II‑induced cardiomyocyte hypertrophy, as evidenced by reduced cell surface area and decreased BNP mRNA levels. Collectively, these results suggest that *CYP4B1* exerts a cardioprotective effect in HF by mitigating pathological hypertrophy. The discrepancy between our results and those of Han et al. may reflect differences in disease stage. In their compensated hypertrophy model, cellular adaptations preserve cardiac output despite increased workload. In contrast, our decompensated hypertrophy model represents the shift to maladaptive remodeling, characterized by reduced contractility and overt heart failure symptoms. These distinct phenotypes likely engage divergent molecular pathways [32], and dissecting their mechanistic underpinnings represents an important avenue for future research.

Previous studies have identified nuclear receptor binding sequences within the *CYP4B1* promoter, including motifs for HIF‑1α, AP-1, and NF-κB [20]. Notably, HIF‑1α is rapidly stabilized under hypoxia, leading to transcriptional upregulation of *CYP4B1* [20]. Additionally, aryl hydrocarbon receptor and HIF-1α agonists have been shown to promote *CYP4B1* expression in high-density mammary cells through NF-κB activation [33]. Despite detailed knowledge of promoter‑bound factors regulating *CYP4B1*, the involvement of distal regulatory elements in fine‑tuning its expression has not yet been addressed. In this study, we identified a distal enhancer, 1p‑Enh, positioned adjacent to the *CYP4B1* 3′‑UTR, indicating that *CYP4B1* is a direct target of this enhancer. 3C assays demonstrated that 1p‑Enh engages the *CYP4B1* promoter via chromatin looping, a mechanism essential for *CYP4B1* transcriptional regulation. Furthermore, luciferase reporter assays confirmed robust 1p‑Enh activity in AC‑16 cardiomyocytes and Beas‑2B bronchial epithelial cells. However, the activity of 1p-Enh was significantly reduced in Ang Ⅱ-induced hypertrophic cardiomyocytes and the A549 and H1703 lung cancer cell lines. Together, these findings not only establish 1p‑Enh as a critical upstream regulator of *CYP4B1* but also reveal its dysregulation as a mechanistic link between heart failure and lung cancer pathogenesis.

Emerging evidence highlights that aberrant activation of the STAT3 signaling plays a critical role in both cardiac hypertrophy and cancer progression, acting as a shared driver of the pathological processes in HF and tumorigenesis [34]. STAT3, a key transcription factor in this pathway, is often hyperactivated in disease states, contributing to maladaptive cardiac remodeling, chronic inflammation, and cellular proliferation [35]. In HF, STAT3 activation promotes cardiomyocyte hypertrophy and fibrosis, whereas in cancer, it drives tumor growth, invasion, and resistance to apoptosis [36]. In agreement with the above reports, our study found that *CYP4B1* exerts protective effects through suppression of the STAT3 phosphorylation in both HF and LC, suggesting the therapeutic potential of targeting CYP4B1/STAT3 pathway for the management of these two conditions.

Smoking is a well-established environmental risk factor for both HF and LC [37–40]. In addition to promoting respiratory malignancies, tobacco smoke also stimulates the renin–angiotensin system [41], and exacerbates cardiovascular injury through oxidative stress, endothelial dysfunction, and chronic inflammation [42]. In our experiments, AC‑16 cardiomyocytes and Beas‑2B bronchial epithelial cells exposed to the tobacco‐specific carcinogen NNK underwent concordant changes in 1p‑Enh activity and *CYP4B1* expression. Specifically, increased 1p‑Enh activity correlated with elevated *CYP4B1* expression, whereas reduced enhancer activity led to decreased transcript levels, implying a direct regulatory link between 1p‑Enh and its target gene. Given that aberrant 1p‑Enh activity and *CYP4B1* expression are implicated in both cardiac remodeling and tumorigenesis, these findings suggest that smoking-induced dysregulation of the 1p‑Enh/CYP4B1 axis may contribute to HF–LC comorbidity. Importantly, epidemiological evidence indicates that smoking cessation significantly decreases the incidence of HF and LC [43,44], highlighting cessation as a critical preventive strategy to restore normal enhancer function and mitigate comorbidity risk.

Our experiments establish *CYP4B1* as a tumor suppressor in LC and a mitigator of pathological remodeling in HF. However, a limitation of our study is the lack of detailed clinical covariates, such as treatment histories and additional comorbidities in the GEO and TCGA datasets, which may influence *CYP4B1* expression and its biomarker performance. Future work should leverage cohorts with richer clinical annotation or prospective designs to assess the impact of specific therapies (e.g., angiotensin‑modulating drugs, chemotherapy regimens) and coexisting conditions on the 1p-Enh/*CYP4B1* axis. Crucially, direct evidence from enhancer mutation or knockout experiments is needed to confirm the essential role of 1p-Enh. Specifically, CRISPR/Cas9‑mediated deletion of 1p‑Enh should also be performed to validate its contribution to *CYP4B1* transcriptional regulation. In parallel, ChIP‑Seq profiling of 1p‑Enh should be undertaken to identify bound transcription factors and elucidate their roles in *CYP4B1* regulation. Beyond its role as a biomarker, *CYP4B1* represents a tractable therapeutic target in both HF and LC. Small‑molecule screens could identify agonists that enhance CYP4B1’s enzymatic activity to suppress STAT3 signaling. Alternatively, epigenetic drugs or CRISPR‑based approaches targeting 1p‑Enh may restore *CYP4B1* expression in disease contexts. Moreover, integrating multi‑omics datasets, as emphasized by recent bibliometric analyses [45], can accelerate the discovery and optimization of such *CYP4B1* modulators for dual‑pathology intervention.

## Conclusions

We identified a novel smoking‑induced 1p‑Enh/*CYP4B1* regulatory axis that mechanistically links tobacco exposure to the comorbid development of HF and LC. Modulating 1p-Enh or restoring CYP4B1 expression pharmacologically may offer a novel strategy to simultaneously reduce the risk and progression of both diseases in at-risk populations.

## Supporting information

S1 Fig GSEA enrichment analysis showing the top pathways enriched in HF samples, providing insight into functional shifts at the systems level. **(A)** GSEA-based enrichment analysis of Gene Ontology (GO) terms. **(B)** GSEA identifies significantly enriched KEGG pathways. **(C)** Reactome pathway enrichment analysis using GSEA.
(DOCX)

S2 Fig Results of the Weighted gene coexpression network analysis (WGCNA) in the GSE141910 dataset. **(A)** The soft threshold power of WGCNA in GSE141910. (**B)** The sample clustering to detect outliers. **(C)** The merged graphical result shows the final clustering of samples under different network modules.
(DOCX)

S3 Fig Results of the Weighted gene coexpression network analysis (WGCNA) in the GSE57338 dataset. **(A)** The soft threshold power of WGCNA in GSE57338. **(B)** The sample clustering to detect outliers. **(C)** The merged graphical result shows the final clustering of samples under different network modules.
(DOCX)

S4 Fig qRT-PCR analysis of STAT3 downstream targets gene expression. (A) Ang II stimulation upregulates SOCS3 and TGF-β1 in AC-16 cardiomyocytes. (B) CYP4B1 overexpression suppresses SOCS3 and TGF-β1 expression in AC-16 cells. (C) CYP4B1 overexpression inhibits CCND1 and TGF-β1 expression in A549 lung cancer cells. (D) CYP4B1 overexpression inhibits CCND1 and TGF-β1 expression in H1703 lung cancer cells. Data are shown as mean ± SD from three independent experiments. *P < 0.05, **P < 0.01 versus control.
(DOCX)

S5 Fig The prediction of CpG island in CYP4B1 promoter follows the criteria: Island size > 100, GC Percent > 50.0, Obs/Exp > 0.60, and no CpG islands were found in CYP4B1 promoter sequence. (DOCX)

S1 Table The top 10 GO terms of the DEGs in BP, CC and MF. (DOCX)

S2 Table The significant pathway in KEGG enrichment. (DOCX)

S3 Table The top enriched terms for GSEA analysis. (DOCX)

S4 Table 70 candidate genes were identified from the intersection of DEGs and WGCNA. (DOCX)

S5 Table The lambda_min results based on 70 candidate genes. (DOCX)

S6 Table The primer sequence of RT-PCR used in this study. (DOCX)

S7 Table The primer sequence of 3C experiments used in this study. (DOCX)
