# Supplementary material for: 1p-Enh-regulated CYP4B1 alleviates NNK-induced heart failure and lung cancer via the STAT3 pathway
Source: PLoS One. 2025 Sep 9;20(9):e0331471. doi: 10.1371/journal.pone.0331471 (PMC12419636; doi:10.1371/journal.pone.0331471)
Supplement: S1 Fig — (A) GSEA-based enrichment analysis of Gene Ontology (GO) terms. (B) GSEA identifies significantly enriched KEGG pathways. (C) Reactome pathway enrichment analysis using GSEA. (DOCX) [file pone.0331471.s001.docx]

**Figure S1**


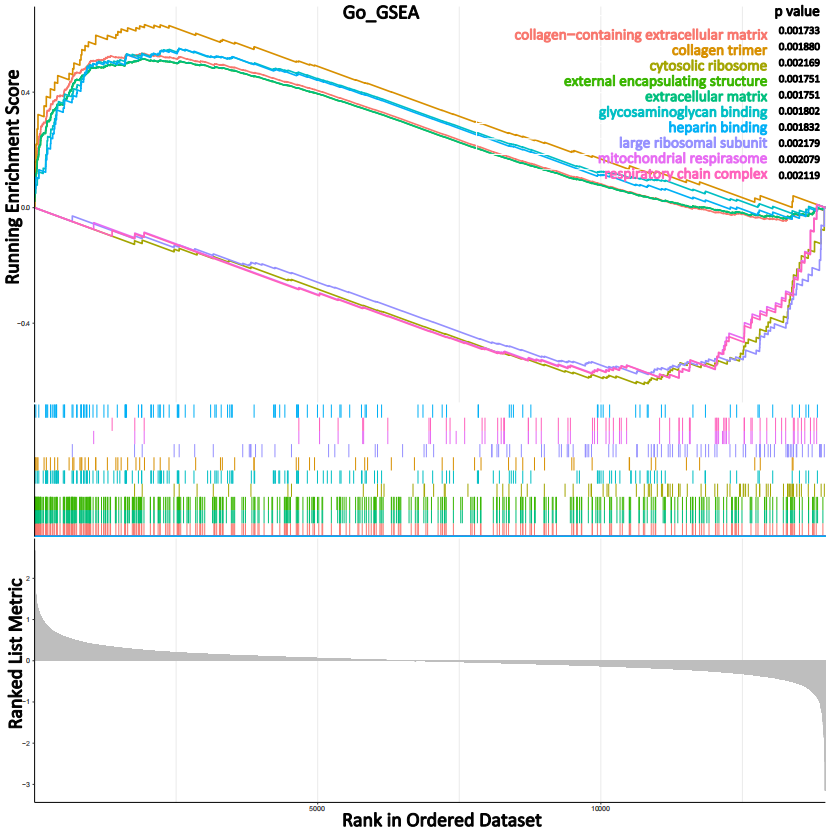


**A**


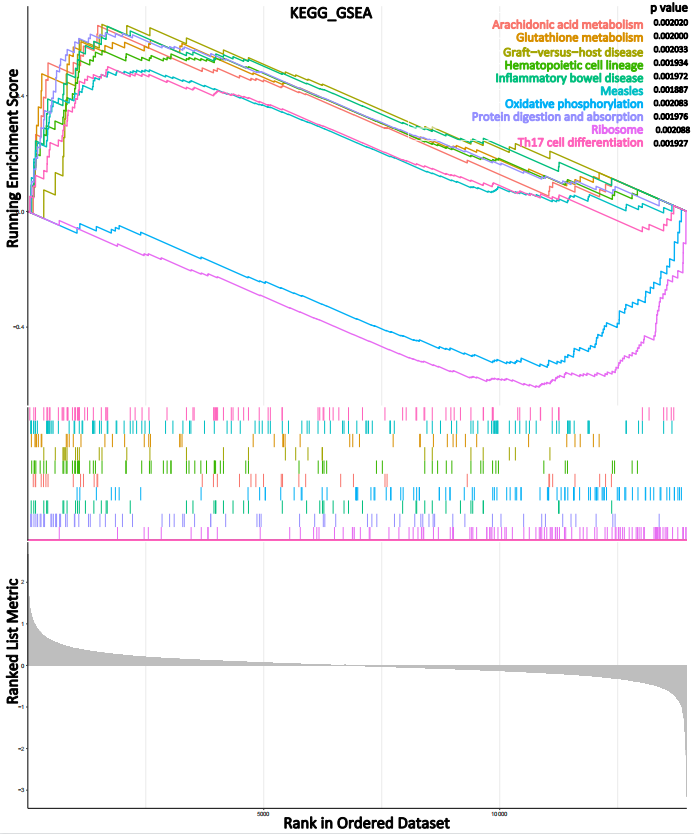


**B**


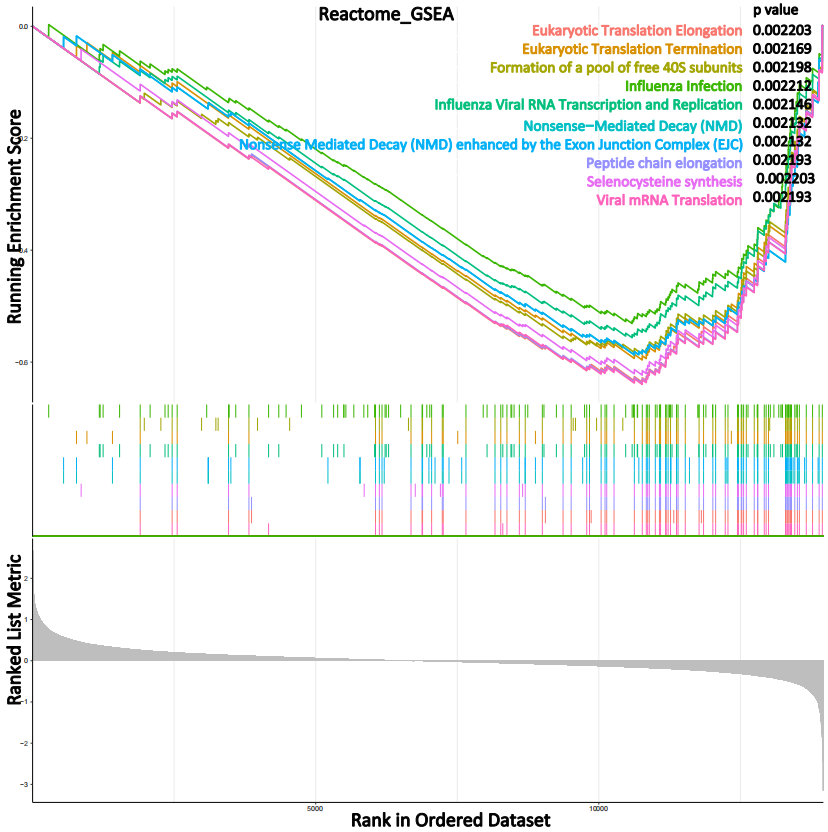


**C**

**S1 Fig. GSEA enrichment analysis showing the top pathways enriched in HF samples, providing insight into functional shifts at the systems level. (A)** GSEA-based enrichment analysis of Gene Ontology (GO) terms. **(B)** GSEA identifies significantly enriched KEGG pathways. **(C)** Reactome pathway enrichment analysis using GSEA.
