# Supplementary material for: 1p-Enh-regulated CYP4B1 alleviates NNK-induced heart failure and lung cancer via the STAT3 pathway
Source: PLoS One. 2025 Sep 9;20(9):e0331471. doi: 10.1371/journal.pone.0331471 (PMC12419636; doi:10.1371/journal.pone.0331471)
Supplement: S2 Fig — (A) The soft threshold power of WGCNA in GSE141910. (B) The sample clustering to detect outliers. (C) The merged graphical result shows the final clustering of samples under different network modules. (DOCX) [file pone.0331471.s002.docx]

**Figure S2**


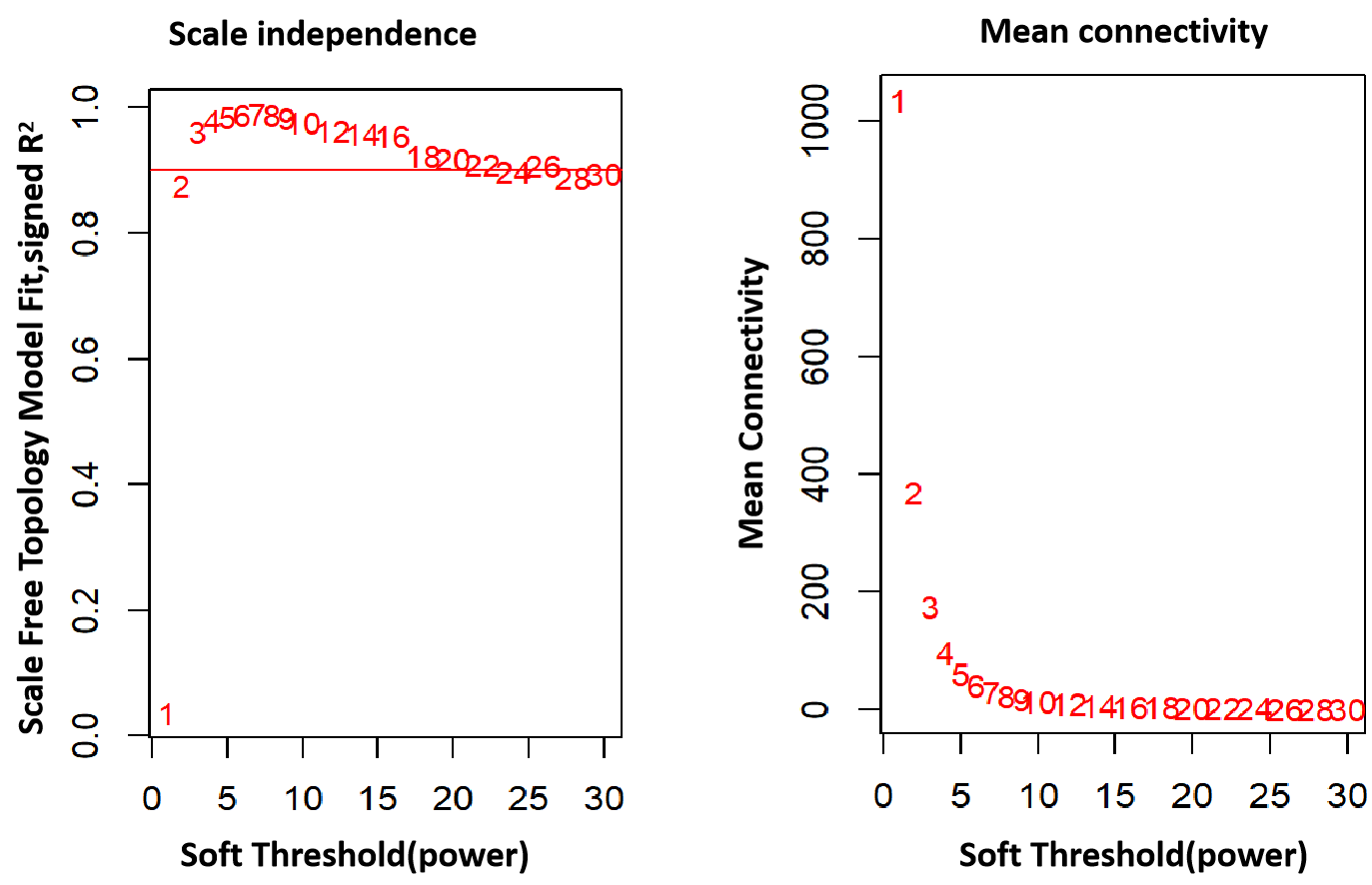


**A**


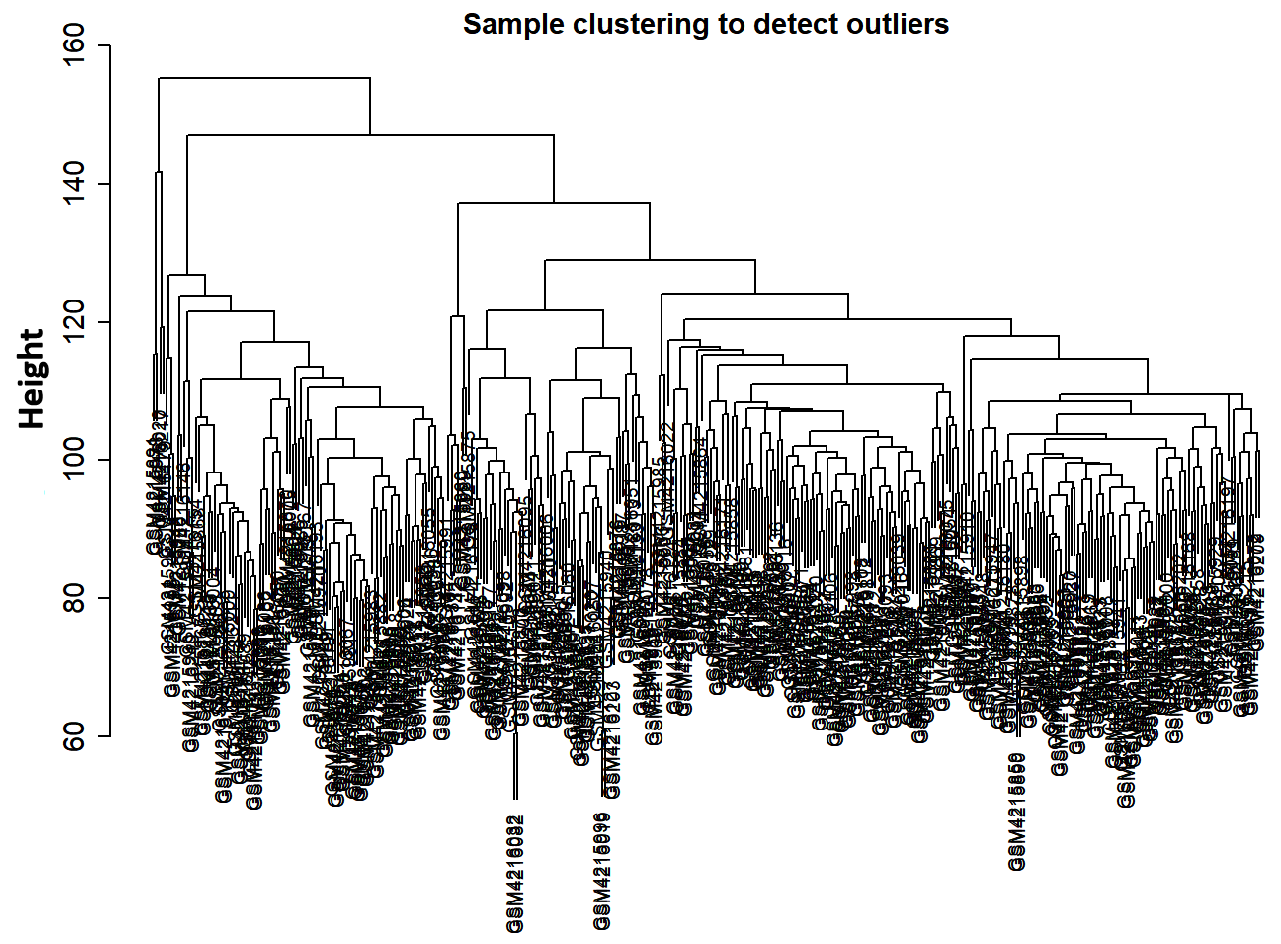


**B**


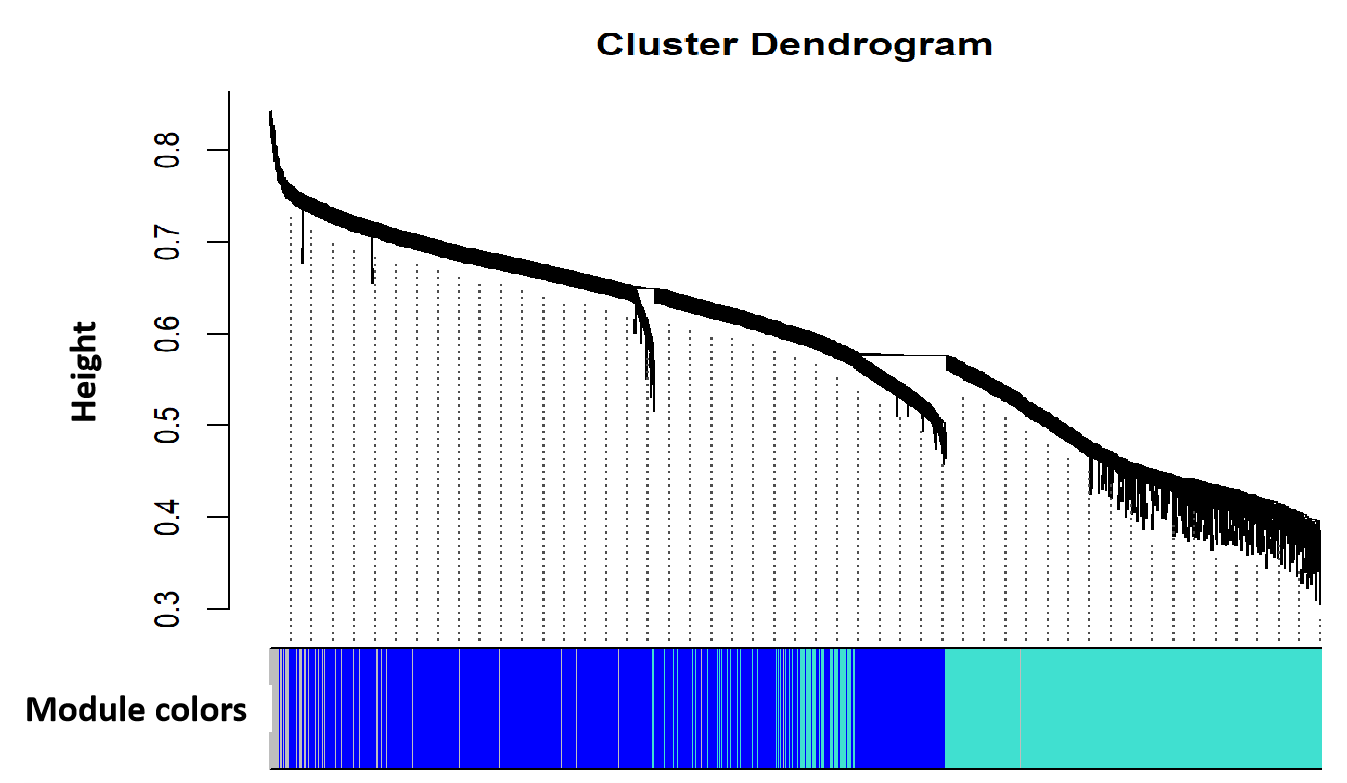


**C**

**S2 Fig. Results of the Weighted gene coexpression network analysis (WGCNA) in the GSE141910 dataset. (A)** The soft threshold power of WGCNA in GSE141910. (**B)** The sample clustering to detect outliers. **(C)** The merged graphical result shows the final clustering of samples under different network modules.
