# Supplementary material for: 1p-Enh-regulated CYP4B1 alleviates NNK-induced heart failure and lung cancer via the STAT3 pathway
Source: PLoS One. 2025 Sep 9;20(9):e0331471. doi: 10.1371/journal.pone.0331471 (PMC12419636; doi:10.1371/journal.pone.0331471)
Supplement: S4 Fig — (A) Ang II stimulation upregulates SOCS3 and TGF-β1 in AC-16 cardiomyocytes. (B) CYP4B1 overexpression suppresses SOCS3 and TGF-β1 expression in AC-16 cells. (C) CYP4B1 overexpression inhibits CCND1 and TGF-β1 expression in A549 lung cancer cells. (D) CYP4B1 overexpression inhibits CCND1 and TGF-β1 expression in H1703 lung cancer cells. Data are shown as mean ± SD from three independent experiments. *P < 0.05, **P < 0.01 versus control. (DOCX) [file pone.0331471.s004.docx]

**Figure S4**


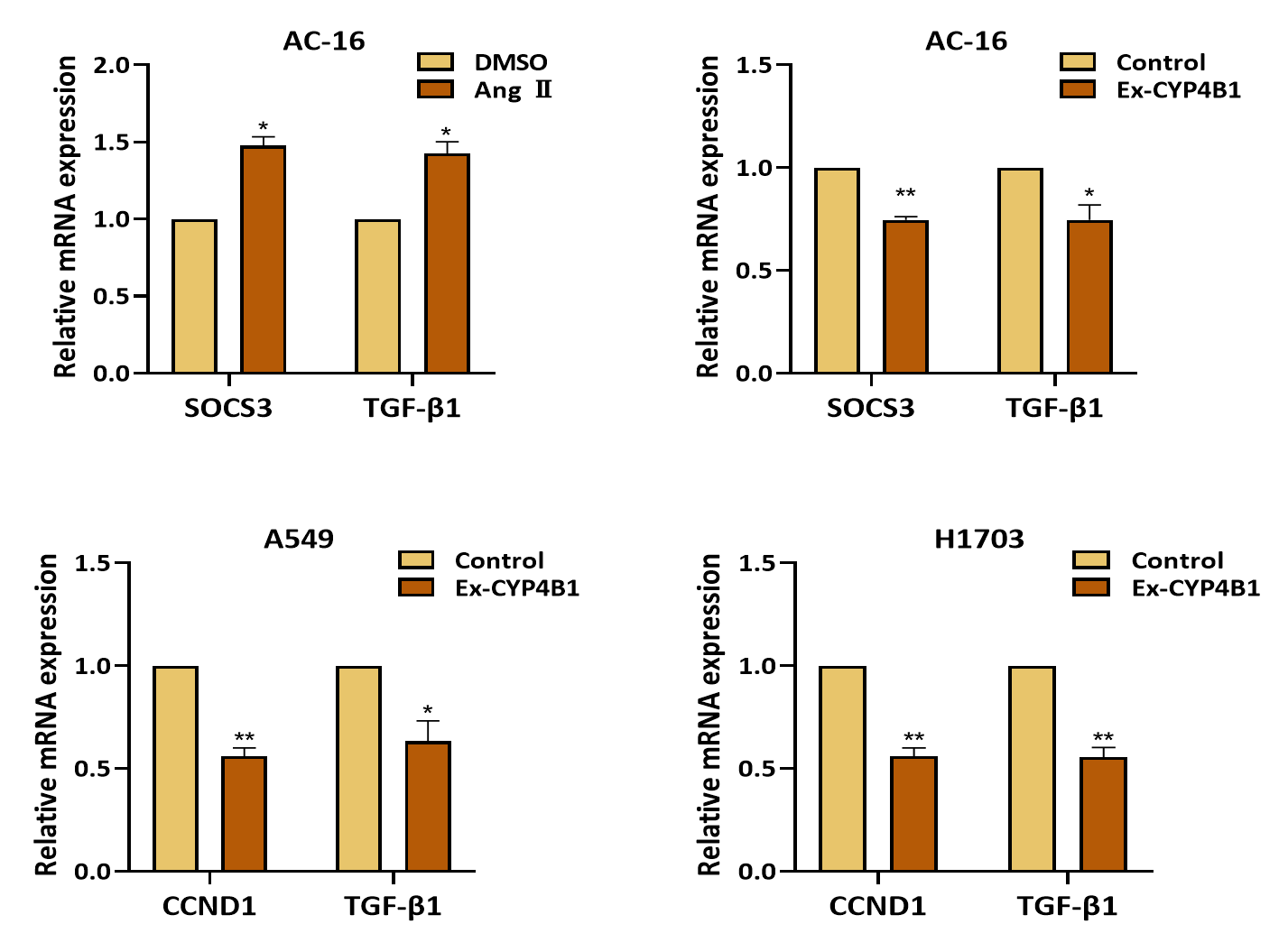


**D**

**C**

**B**

**A**

**S4 Fig. qRT-PCR analysis of STAT3 downstream targets gene expression. (A)** Ang II stimulation upregulates SOCS3 and TGF‑β1 in AC‑16 cardiomyocytes. **(B)** CYP4B1 overexpression suppresses SOCS3 and TGF‑β1 expression in AC‑16 cells. **(C)** CYP4B1 overexpression inhibits CCND1 and TGF‑β1 expression in A549 lung cancer cells. **(D)** CYP4B1 overexpression inhibits CCND1 and TGF‑β1 expression in H1703 lung cancer cells. Data are shown as mean ± SD from three independent experiments. *P < 0.05, **P < 0.01 versus control.
