# Supplementary material for: 1p-Enh-regulated CYP4B1 alleviates NNK-induced heart failure and lung cancer via the STAT3 pathway
Source: PLoS One. 2025 Sep 9;20(9):e0331471. doi: 10.1371/journal.pone.0331471 (PMC12419636; doi:10.1371/journal.pone.0331471)
Supplement: S5 Fig — (DOCX) [file pone.0331471.s005.docx]

**Figure S5**


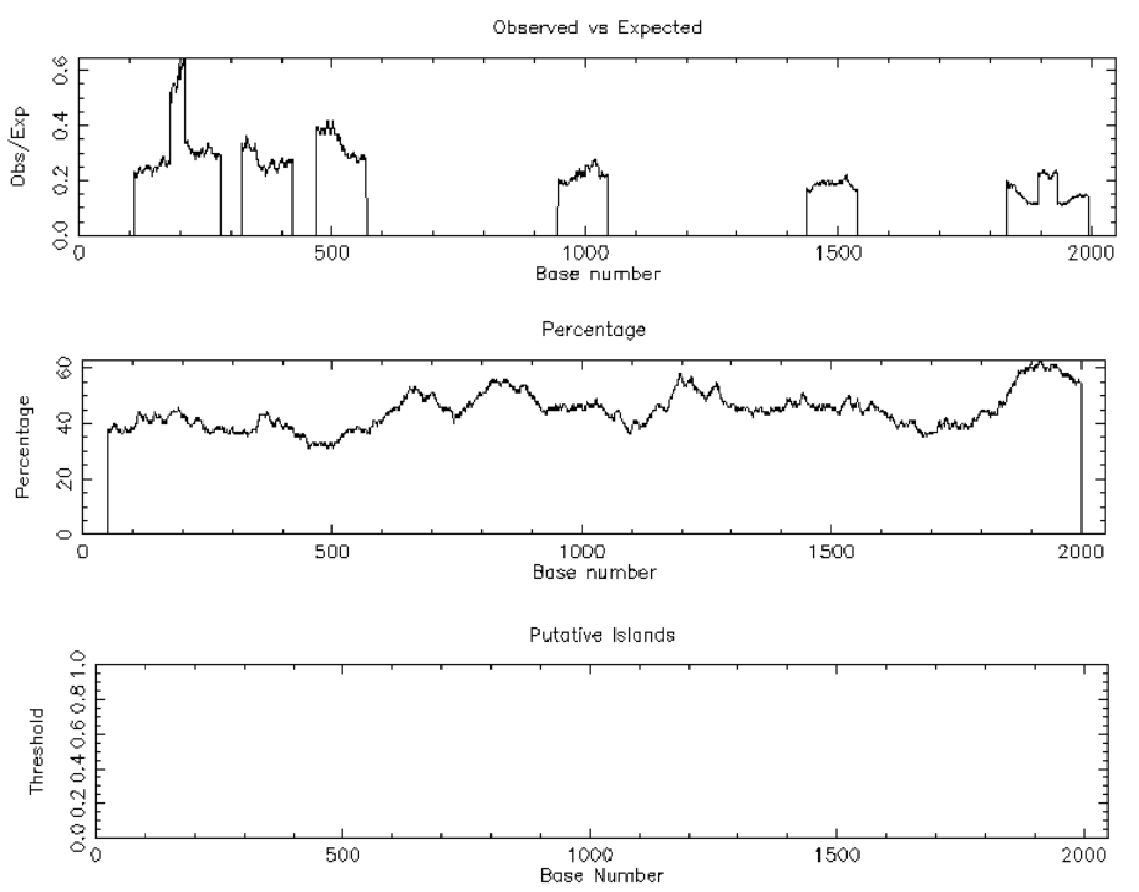


**S5 Fig.** The prediction of CpG island in CYP4B1 promoter follows the criteria: Island size > 100, GC Percent > 50.0, Obs/Exp > 0.60, and no CpG islands were found in CYP4B1 promoter sequence.
