# Supplementary material for: 1p-Enh-regulated CYP4B1 alleviates NNK-induced heart failure and lung cancer via the STAT3 pathway
Source: PLoS One. 2025 Sep 9;20(9):e0331471. doi: 10.1371/journal.pone.0331471 (PMC12419636; doi:10.1371/journal.pone.0331471)
Supplement: S1 Table — (DOCX) [file pone.0331471.s006.docx]

**Table.S1 The top 10 GO terms of the DEGs in BP, CC and MF**

| **ONTOLOGY** | **ID** | **Description** | **pvalue** |
| --- | --- | --- | --- |
| BP | GO:0030198 | extracellular matrix organization | 9.65E-11 |
| BP | GO:0043062 | extracellular structure organization | 1.04E-10 |
| BP | GO:0045229 | external encapsulating structure organization | 1.19E-10 |
| BP | GO:0045765 | regulation of angiogenesis | 6.77E-09 |
| BP | GO:1901342 | regulation of vasculature development | 9.50E-09 |
| BP | GO:0009636 | response to toxic substance | 3.17E-08 |
| BP | GO:0060326 | cell chemotaxis | 1.11E-07 |
| BP | GO:0042060 | wound healing | 3.55E-07 |
| BP | GO:0072503 | cellular divalent inorganic cation homeostasis | 3.71E-07 |
| BP | GO:0043062 | extracellular structure organization | 1.04E-10 |
| CC | GO:0062023 | collagen-containing extracellular matrix | 2.12E-29 |
| CC | GO:0005604 | basement membrane | 8.78E-08 |
| CC | GO:0005581 | collagen trimer | 2.31E-06 |
| CC | GO:0072562 | blood microparticle | 8.07E-06 |
| CC | GO:0009897 | external side of plasma membrane | 1.28E-05 |
| CC | GO:0016529 | sarcoplasmic reticulum | 0.000190086 |
| CC | GO:0005775 | vacuolar lumen | 0.000207956 |
| CC | GO:0043202 | lysosomal lumen | 0.00025778 |
| CC | GO:0030667 | secretory granule membrane | 0.000318513 |
| CC | GO:0016528 | sarcoplasm | 0.000403279 |
| MF | GO:0005201 | extracellular matrix structural constituent | 4.33E-14 |
| MF | GO:0005539 | glycosaminoglycan binding | 2.06E-11 |
| MF | GO:1901681 | sulfur compound binding | 1.08E-08 |
| MF | GO:0008201 | heparin binding | 4.20E-08 |
| MF | GO:0030021 | extracellular matrix structural constituent conferring compression resistance | 6.77E-08 |
| MF | GO:0005518 | collagen binding | 3.44E-07 |
| MF | GO:0140375 | immune receptor activity | 1.67E-06 |
| MF | GO:0050135 | NAD(P)+ nucleosidase activity | 6.17E-06 |
| MF | GO:0061809 | NAD+ nucleotidase, cyclic ADP-ribose generating | 6.17E-06 |
| MF | GO:0005178 | integrin binding | 9.59E-06 |
