# Supplementary material for: 1p-Enh-regulated CYP4B1 alleviates NNK-induced heart failure and lung cancer via the STAT3 pathway
Source: PLoS One. 2025 Sep 9;20(9):e0331471. doi: 10.1371/journal.pone.0331471 (PMC12419636; doi:10.1371/journal.pone.0331471)
Supplement: S2 Table — (DOCX) [file pone.0331471.s007.docx]

**Table.S2 The significant pathway in KEGG enrichment**

| **ID** | **Description** | **pvalue** |
| --- | --- | --- |
| hsa04933 | AGE-RAGE signaling pathway in diabetic complications | 1.37E-06 |
| hsa05144 | Malaria | 8.64E-05 |
| hsa04610 | Complement and coagulation cascades | 8.89E-05 |
| hsa04145 | Phagosome | 0.000100185 |
| hsa04640 | Hematopoietic cell lineage | 0.00026343 |
| hsa04919 | Thyroid hormone signaling pathway | 0.000264211 |
| hsa04978 | Mineral absorption | 0.000278684 |
| hsa05142 | Chagas disease | 0.000329854 |
| hsa05321 | Inflammatory bowel disease | 0.000459031 |
| hsa05145 | Toxoplasmosis | 0.000616831 |
| hsa00982 | Drug metabolism - cytochrome P450 | 0.000856222 |
| hsa00760 | Nicotinate and nicotinamide metabolism | 0.001082848 |
