# Supplementary material for: 1p-Enh-regulated CYP4B1 alleviates NNK-induced heart failure and lung cancer via the STAT3 pathway
Source: PLoS One. 2025 Sep 9;20(9):e0331471. doi: 10.1371/journal.pone.0331471 (PMC12419636; doi:10.1371/journal.pone.0331471)
Supplement: S3 Table — (DOCX) [file pone.0331471.s008.docx]

**Table.S3 The top enriched terms for GSEA analysis**

| **ID** | **Description** | **NES** | **pvalue** |
| --- | --- | --- | --- |
| GO:0062023 | collagen-containing extracellular matrix | 2.318054008 | 0.001733102 |
| GO:0031012 | extracellular matrix | 2.28143161 | 0.001751313 |
| GO:0030312 | external encapsulating structure | 2.280285367 | 0.001751313 |
| GO:0022626 | cytosolic ribosome | -2.244926706 | 0.002169197 |
| GO:0005539 | glycosaminoglycan binding | 2.225240652 | 0.001801802 |
| GO:0005581 | collagen trimer | 2.213131428 | 0.001879699 |
| GO:0015934 | large ribosomal subunit | -2.180447297 | 0.002178649 |
| GO:0005746 | mitochondrial respirasome | -2.136489159 | 0.002079002 |
| GO:0098803 | respiratory chain complex | -2.116698722 | 0.002118644 |
| GO:0008201 | heparin binding | 2.1136222 | 0.001831502 |
| R-HSA-192823 | Viral mRNA Translation | -2.285304578 | 0.002192982 |
| R-HSA-156842 | Eukaryotic Translation Elongation | -2.282454611 | 0.002202643 |
| R-HSA-156902 | Peptide chain elongation | -2.278915876 | 0.002192982 |
| R-HSA-2408557 | Selenocysteine synthesis | -2.236870366 | 0.002202643 |
| R-HSA-927802 | Nonsense-Mediated Decay (NMD) | -2.213696831 | 0.002132196 |
| R-HSA-975957 | Nonsense Mediated Decay (NMD) enhanced by the Exon Junction Complex (EJC) | -2.213696831 | 0.002132196 |
| R-HSA-168273 | Influenza Viral RNA Transcription and Replication | -2.157846209 | 0.002145923 |
| R-HSA-72764 | Eukaryotic Translation Termination | -2.150643462 | 0.002169197 |
| R-HSA-72689 | Formation of a pool of free 40S subunits | -2.148963112 | 0.002197802 |
| R-HSA-168255 | Influenza Infection | -2.110657882 | 0.002212389 |
| hsa03010 | Ribosome | -2.37371439 | 0.002087683 |
| hsa04974 | Protein digestion and absorption | 2.165123095 | 0.001976285 |
| hsa05321 | Inflammatory bowel disease | 2.11693952 | 0.001972387 |
| hsa00190 | Oxidative phosphorylation | -2.048635348 | 0.002083333 |
| hsa00590 | Arachidonic acid metabolism | 1.984549993 | 0.002020202 |
| hsa04640 | Hematopoietic cell lineage | 1.947242743 | 0.001934236 |
| hsa05332 | Graft-versus-host disease | 1.933782729 | 0.00203252 |
| hsa00480 | Glutathione metabolism | 1.920625242 | 0.002 |
| hsa05162 | Measles | 1.868096799 | 0.001886792 |
| hsa04659 | Th17 cell differentiation | 1.866623064 | 0.001926782 |
