# Supplementary material for: 1p-Enh-regulated CYP4B1 alleviates NNK-induced heart failure and lung cancer via the STAT3 pathway
Source: PLoS One. 2025 Sep 9;20(9):e0331471. doi: 10.1371/journal.pone.0331471 (PMC12419636; doi:10.1371/journal.pone.0331471)
Supplement: S4 Table — (DOCX) [file pone.0331471.s009.docx]

**Table.S4 70 candidate genes were identified from the intersection of DEGs and WGCNA module genes**

| **List** | **Gene** | **List** | **Gene** | **List** | **Gene** | **List** | **Gene** |
| --- | --- | --- | --- | --- | --- | --- | --- |
| 1 | TUBA3D | 19 | SCUBE2 | 37 | SULF1 | 55 | STAT4 |
| 2 | FREM1 | 20 | LCN10 | 38 | DIO2 | 56 | APLNR |
| 3 | FCN3 | 21 | IFI44L | 39 | CHDH | 57 | CCDC80 |
| 4 | SMOC2 | 22 | AQP3 | 40 | RASL11B | 58 | CA14 |
| 5 | LAD1 | 23 | MYH6 | 41 | RGS4 | 59 | ATP1B4 |
| 6 | ECM2 | 24 | PHLDA1 | 42 | GATM | 60 | MYOC |
| 7 | PDE5A | 25 | COL14A1 | 43 | SHISA3 | 61 | FAP |
| 8 | LUM | 26 | PLEKHH2 | 44 | MOXD1 | 62 | FAM155B |
| 9 | ASPN | 27 | MME | 45 | ITGBL1 | 63 | COLQ |
| 10 | FRZB | 28 | MFAP4 | 46 | CRHBP | 64 | F2RL2 |
| 11 | NRK | 29 | CRISPLD1 | 47 | HAPLN1 | 65 | POSTN |
| 12 | MATN2 | 30 | MXRA5 | 48 | CBS | 66 | P3H2 |
| 13 | TUBA3E | 31 | NT5E | 49 | CX3CR1 | 67 | CXCL14 |
| 14 | FNDC1 | 32 | ACKR4 | 50 | LTBP2 | 68 | TNC |
| 15 | PTN | 33 | METTL7B | 51 | FMOD | 69 | DHRS7C |
| 16 | SFRP4 | 34 | LRRC17 | 52 | THBS4 | 70 | STAT4 |
| 17 | C1QTNF7 | 35 | ANKRD2 | 53 | EDA2R |  |  |
| 18 | OGN | 36 | CYP4B1 | 54 | NRG1 |  |  |
