# Supplementary material for: 1p-Enh-regulated CYP4B1 alleviates NNK-induced heart failure and lung cancer via the STAT3 pathway
Source: PLoS One. 2025 Sep 9;20(9):e0331471. doi: 10.1371/journal.pone.0331471 (PMC12419636; doi:10.1371/journal.pone.0331471)
Supplement: S5 Table — (DOCX) [file pone.0331471.s010.docx]

**Table.S5 The lambda_min results based on 70 candidate genes**

| **Gene** | **Coef value** |
| --- | --- |
| MYOC | -0.56336051 |
| FAP | 0.11985539 |
| P3H2 | -0.52602827 |
| ACKR4 | -0.46067964 |
| FAM155B | 0.22617093 |
| FCN3 | -0.06605593 |
| CYP4B1 | -0.04852222 |
| HAPLN1 | 0.03764124 |
| NRG1 | -0.24575403 |
| LAD1 | 0.19037792 |
| ANKRD2 | -0.05013575 |
| NTM | -0.02306495 |
| MME | -0.73143962 |
| DIO2 | 0.02367154 |
