# Supplementary material for: 1p-Enh-regulated CYP4B1 alleviates NNK-induced heart failure and lung cancer via the STAT3 pathway
Source: PLoS One. 2025 Sep 9;20(9):e0331471. doi: 10.1371/journal.pone.0331471 (PMC12419636; doi:10.1371/journal.pone.0331471)
Supplement: S6 Table — (DOCX) [file pone.0331471.s011.docx]

**Table.S6 The primer sequence of RT-PCR used in this study**

| **Gene** | **Primer sequence** |
| --- | --- |
| ANP | F: CAACGCAGACCTGATGGATTT  R: AGCCCCCGCTTCTTCATTC |
| BNP | F: TGGAAACGTCCGGGTTACAG  R: CTGATCCGGTCCATCTTCCT |
| β-MyHC | F: TGGTTTTGAGGCAGCTAGTATCA  R: AGTCCTGAATAGTAGCGATCCTT |
| COL1A1 | F: GAGGGCCAAGACGAAGACATC  R: CAGATCACGTCATCGCACAAC |
| CYP4B1 | F: GACGTTGGCTAAGGCTATGGA  R: CTCCCCGTCTCCTGGATCTC |
| ACTB | F: CATGTACGTTGCTATCCAGGC  R: CTCCTTAATGTCACGCACGAT |
| SOCS3 | F:CCTGCGCCTCAAGACCTTC  R:GTCACTGCGCTCCAGTAGAA |
| TGFβ1 | F:GGCCAGATCCTGTCCAAGC  R:GTGGGTTTCCACCATTAGCAC |
| CCND1 | F:GCTGCGAAGTGGAAACCATC  R:CCTCCTTCTGCACACATTTGAA |
