# Supplementary material for: 1p-Enh-regulated CYP4B1 alleviates NNK-induced heart failure and lung cancer via the STAT3 pathway
Source: PLoS One. 2025 Sep 9;20(9):e0331471. doi: 10.1371/journal.pone.0331471 (PMC12419636; doi:10.1371/journal.pone.0331471)
Supplement: S7 Table — (DOCX) [file pone.0331471.s012.docx]

**Table.S7 The primer sequence of 3C experiments used in this study**

| **Primer** | **Sequence** |
| --- | --- |
| Primer A | AATCCAGGACCAGACGGATTCAC |
| Primer B | CATCAACACAGCTGGACTAGAGCAG |
| Primer C | CAGCAGGTCCCTAGTATCAGATAG |
| Primer D | TATCAGGATGATGCTGGCCTCGTA |
| Loading control | F: GAAATCGTGCGTGACATTAA  R: AAGGAAGGCTGGAAGAGTG |
